# Supplementary material for: Downregulation of extraembryonic tension controls body axis formation in avian embryos
Source: Nat Commun. 2023 Jun 5;14:3266. doi: 10.1038/s41467-023-38988-3 (PMC10241863; doi:10.1038/s41467-023-38988-3)
Supplement: Supplementary file 1 — Supplementary Information [file 41467_2023_38988_MOESM1_ESM.pdf]

Supplementary Information

Downregulation of Extraembryonic Tension Controls Body Axis Formation in Avian Embryos

Daniele Kunz[1,2], Anfu Wang[1], Chon U Chan[3], Robyn H. Pritchard[4,5], Wenyu Wang[5], Filomena Gallo[6], Charles R. Bradshaw[1], Elisa Terenzani[1], Karin H. Müller[6], Yan Yan Shery Huang[5], Fengzhu Xiong[1,2,*]

[1]Wellcome Trust / CRUK Gurdon Institute, University of Cambridge, Cambridge, UK
[2]Department of Physiology, Development and Neuroscience, University of Cambridge, Cambridge, UK
[3]Institute of Molecular and Cell Biology, A*STAR, Singapore
[4]Department of Physics, University of Cambridge, Cambridge, UK
[5]Department of Engineering, University of Cambridge, Cambridge, UK
[6]Cambridge Advanced Imaging Centre, University of Cambridge, Cambridge, UK
*correspondence: fx220@cam.ac.uk

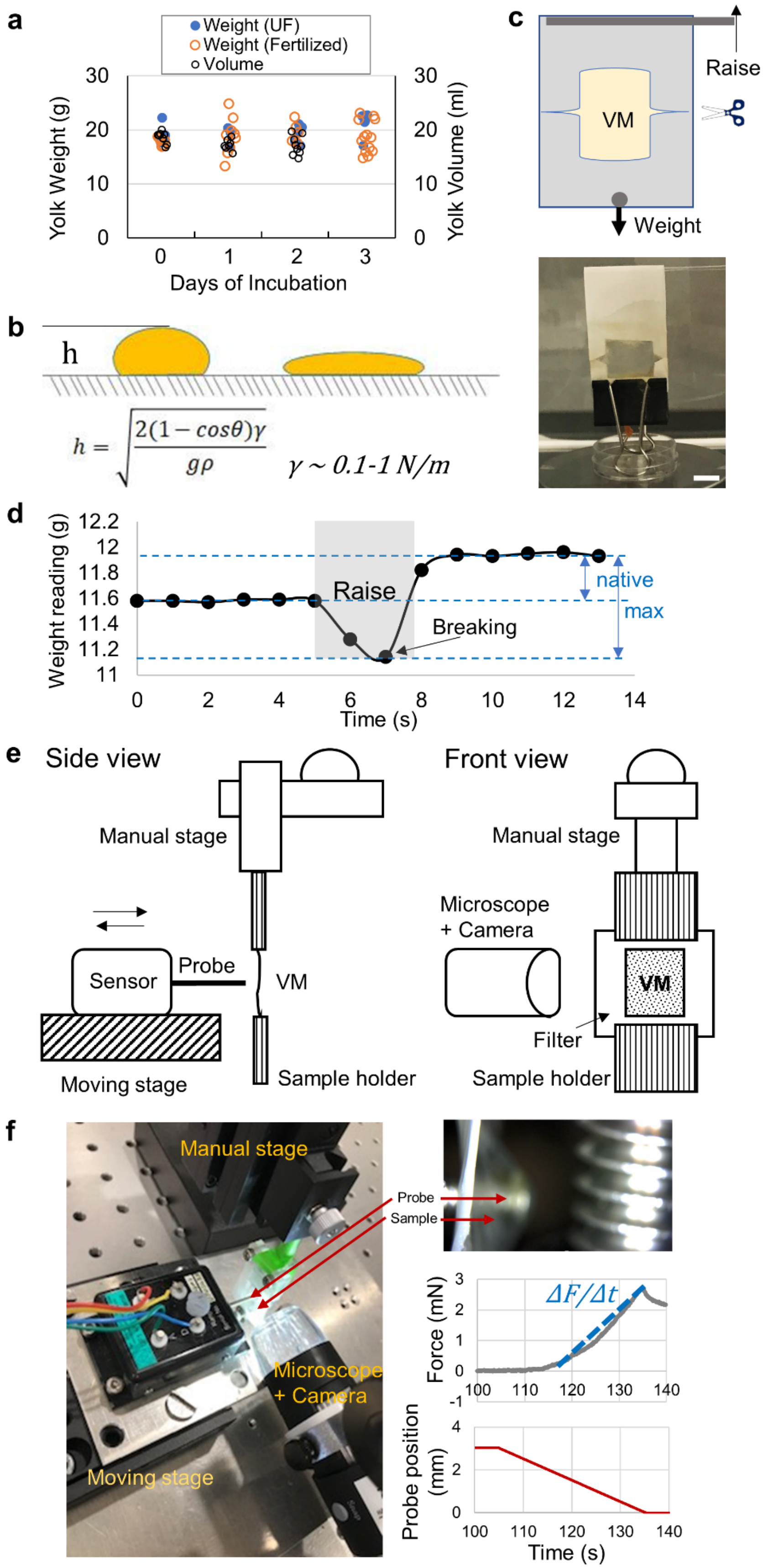
a
Weight (UF)
Weight (Fertilized)
Volume
Yolk Weight (g)
Yolk Volume (ml)
Days of Incubation
c
Raise
VM
Weight
b
h
$h = \sqrt{\frac{2(1-\cos\theta)\gamma}{g\rho}}$
$\gamma \sim 0.1\text{-}1\ N/m$
d
Weight reading (g)
Raise
Breaking
native
max
Time (s)
e
Side view
Manual stage
Sensor
Probe
VM
Moving stage
Sample holder
Front view
Manual stage
Microscope
+ Camera
VM
Filter
Sample holder
f
Manual stage
Microscope
+ Camera
Moving stage
Probe
Sample
$\Delta F/\Delta t$
Force (mN)
Probe position (mm)
Time (s)

Supplementary Figure 1. Physical changes of the yolk and the VM

a. Yolk weight and volume over incubation. Albumen was removed as much as possible for each yolk with a Pasteur pipette before weight measurements. From D0 to D3, n=4,3,5,5 for unfertilized eggs (UF), n=6,9,5,13 for fertilized eggs; For volume measurements, from D0 to D2, n=8,7,9. The weights (p=0.61) and volumes (p=0.06) were similar (One-way ANOVA).

b. Simple droplet model of the yolk shape[1]. h(height)~1-2x10^{-2}m, ρ(density)~1x10^3kg/m^3, g(gravitational acceleration)~10m/s^2, θ(contact angle)>90°. Using our height data (Fig.1c), The VM tension was estimated to be ~1.68 N/m on D0 and ~0.67 N/m on D2.

c. Tension measurement using a scale. Schematic on the left and an example on the right. The VM was sandwiched between two identical sides of a folded piece of windowed filter paper. A clip serving as the weight was attached on the bottom. Small contact points on both sides were designed in the window to minimize side deformation of the VM. After cutting these points the VM suspended the weight and raising the holder increased VM tension. Weight reading was recorded and analyzed in a live video. Scale bar, 1cm.

d. Example of a typical raise experiment. The maximum drop point was identified in the video which usually corresponds to the visible breakage starting to appear. This and the pre-raise line were compared to the stable weight to calculate tensions.

e. Schematic views of the force probe set up. The side view shows the moving stage driving the probe horizontally to contact the center of the mounted VM. The sensor detects a resisting force upon contact. The front view shows the side mini-microscope that captures the contact and deformation dynamics, and the VM sample being mounted on the sample holder.

f. Mechanical property measurement using the probe in panel **e**. The left photo shows the components of the homemade system. Top right shows a measurement in progress from the camera view. The probe is seen to protrude and deform the VM towards the right side away from the filter mount. The plots show an example measurement sequence on a parafilm control. The probe moved at a stable speed and started to detect a force when the sample started to resist deformation. The average rate of increase (mN/s) of a 20s duration over the approximately linear phase of force curve ($\Delta F/\Delta t$ in the plot) was used to measure VM resistance. Note that in this configuration the strain rate of the VM is not constant.

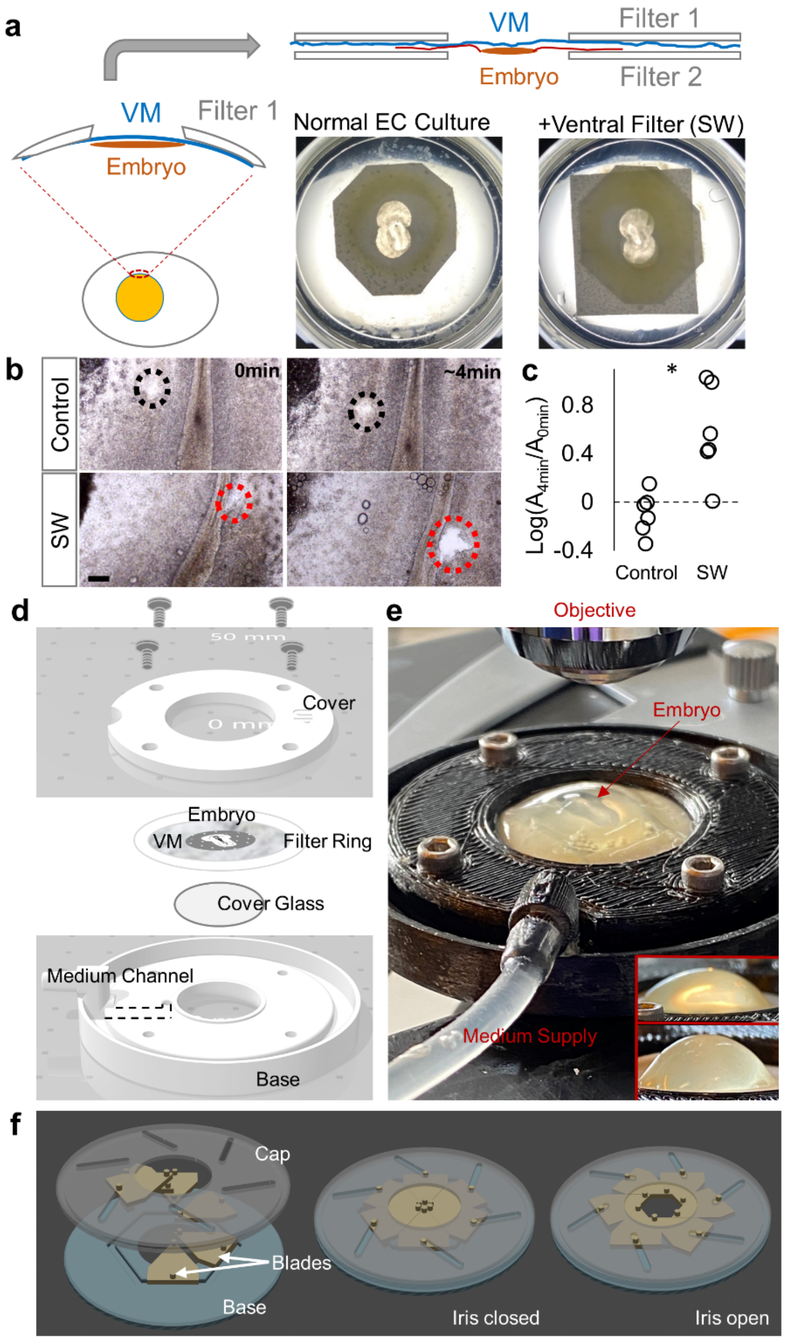

Supplementary Figure 2. Tension perturbations of the VM

a. Filter sandwich (SW) to maintain tension of the VM. In the normal *ex ovo* culture protocol[2], one piece of windowed filter paper (Filter 1) is attached to the VM and the embryo is cut out and laid down on the culture dish with ventral side up. By placing another filter (Filter 2) on the ventral side, the VM and the distal part of the extraembryonic tissue were sandwiched and fixed in place. Tissues in the window (including the embryo) can continue their movements.

b. Wound opening after a needle cut on the embryos. Circles mark the wound site. Scale bar, 100μm.

c. Wound size change. Areas at 0min and approximately 4min after cut are compared. Controls (n=6) show slow opening or closing while SWs (n=6) show consistent expansion. *p=0.003 (2-tailed t-test).

d-e. VM tension modulation with a double-ring inflation culture device. This system is a new design inspired by Sydow et al[3]. The schematic (D) shows the components of the set-up. The photo (E) shows an experiment (Inserts show side views of different degrees of inflation, top is mild, bottom is strong). The cover and base (i.e. the double ring) were 3D printed. Embryo extracted (D1.5) on a filter ring is laid ventral side down on a cover glass which is glued to the opening of the base ring. Screws secure the seal of the sample allowing syringe controlled medium supply to inflate the sample. Samples with damage on the VM causing medium leaking were discarded.

f. 3D rendering of the uniaxial iris stretcher used in Fig.4c. The cap has channels for the blades to slide thereby opening the iris when the cap and base rotate relative to each other. See Source Data for a technical drawing.

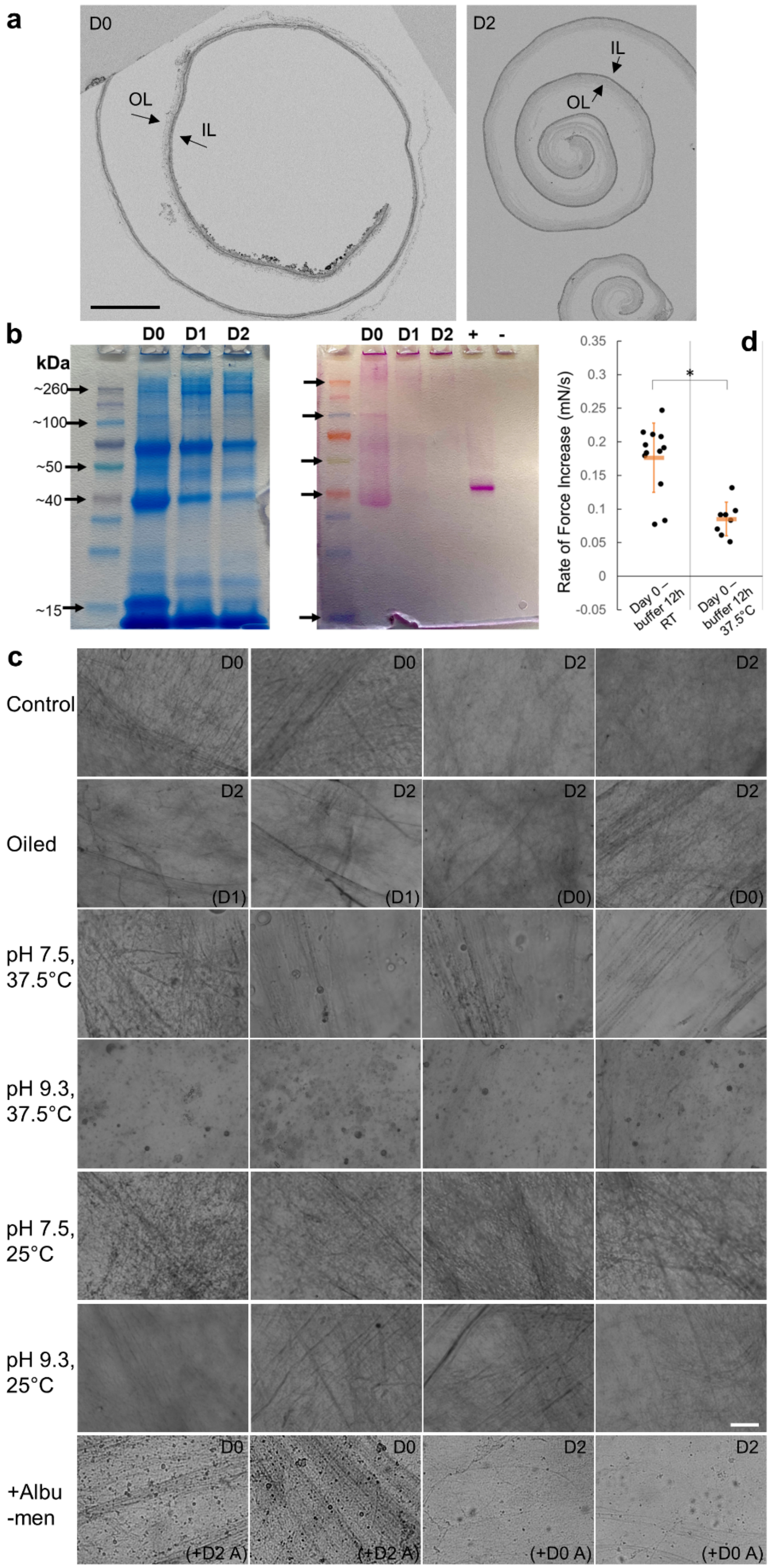

a
D0
OL
IL
D2
IL
OL
b
D0
D1
D2
kDa
~260
~100
~50
~40
~15
D0
D1
D2
+
-
d
Rate of Force Increase (mN/s)
0.35
0.3
0.25
0.2
0.15
0.1
0.05
0
-0.05
*
Day 0 – buffer 12h RT
Day 0 – buffer 12h 37.5°C
c
Control
Oiled
pH 7.5, 37.5°C
pH 9.3, 37.5°C
pH 7.5, 25°C
pH 9.3, 25°C
+Albu -men
D0
D2
(D1)
(D0)
(+D2 A)
(+D0 A)

Supplementary Figure 3. Structural changes of the VM
a. Lower magnification SEM views of the VM cross-sections. OL, outer layer; IL, inner layer. The VM patterns shown in Fig.5a are uniformly found on the samples. Scale bar, 100μm.
b. SDS gels of VM protein extraction stained by Coomassie blue (left) and PAS for glycoproteins (right), respectively, using the same set of samples at normalized concentrations after measuring the optical density with Lowry method. Lysozymes (~14kDa bands at the bottom of the gel) are the main component of the samples. The positive control glycoprotein is Horseradish Peroxidase (~44kDa). Representative of 3 repeats.
c. Glycoprotein fibers on the VM (these are additional examples related to Figs.5b-c,f-i). Top right label shows the Day when the VM is extracted and fixed for PAS staining. Bottom right label shows the Day when oil treatment starts. For the pH buffer treatment (12 hours) groups, all VMs are D0. For the +Albumen samples, bottom right label shows the type of albumen (D0 or D2) added. Scale bar, 10μm. Oil and pH treatments were repeated 3 times.
d. Stiffness measurement of buffer treated VM samples under different temperatures (n=12, RT; n=8, 37°C; p=2e-4, 2-tailed t-test). Bars indicate mean+/-SD.

Supplementary References

1. de Gennes, P. G., Brochard-Wyart, F. & Quéré, D. *Capillarity and wetting phenomena: drops, bubbles, pearls, waves*. (Springer Science & Business Media, 2013).

2. Chapman, S. C., Collignon, J. J., Schoenwolf, G. C. & Lumsden, A. Improved method for chick whole-embryo culture using a filter paper carrier. *Developmental Dynamics* **220**, 284–289 (2001).

3. Sydow, H., Pieper, T., Viebahn, C. & Tsikolia, N. An early Chick embryo culture device for extended continuous observation. in *Avian and Reptilian Developmental Biology* 309–317 (2017).

Intact gels of Fig. S3b

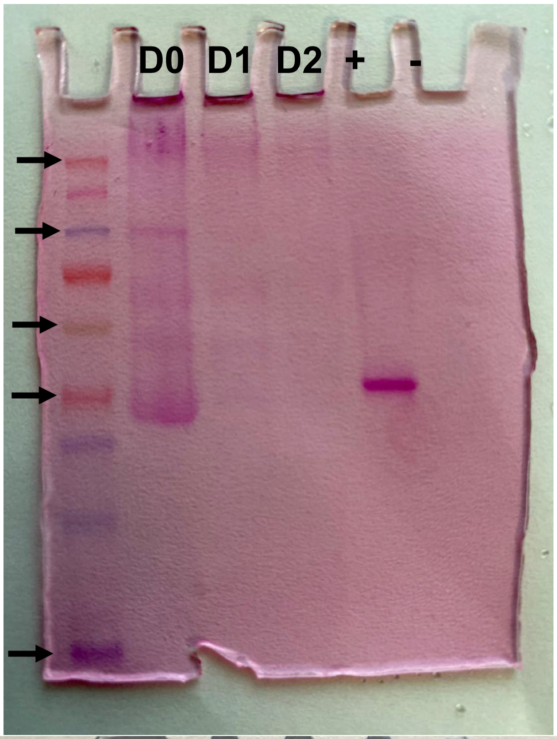
D0 D1 D2 + -

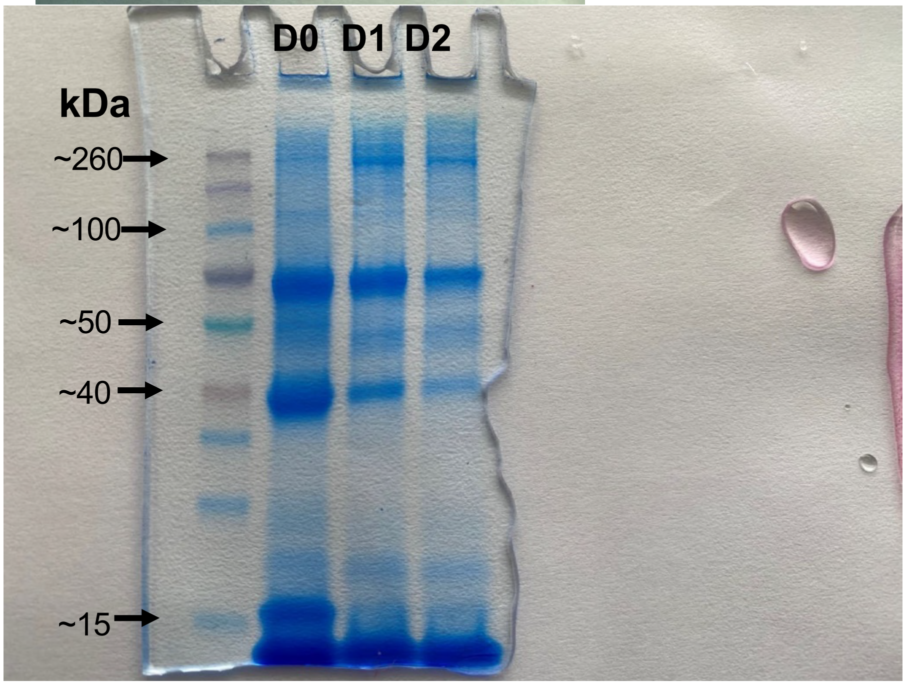
D0 D1 D2
kDa
~260
~100
~50
~40
~15
