# Supplementary material for: Downregulation of extraembryonic tension controls body axis formation in avian embryos
Source: Nat Commun. 2023 Jun 5;14:3266. doi: 10.1038/s41467-023-38988-3 (PMC10241863; doi:10.1038/s41467-023-38988-3)
Supplement: Supplementary file 3 — Description of Additional Supplementary Files [file 41467_2023_38988_MOESM3_ESM.pdf]

**Description of Additional Supplementary Files**

File Name: Supplementary Movie 1
Description: Body axis morphogenesis of a control embryo. Anterior to the left. Consistent posterior body axis narrowing and elongation are visible. Scale bar, 500µm.

File Name: Supplementary Movie 2
Description: Body axis morphogenesis of a SW embryo. Anterior to the left. The embryo is visibly shorter and wider. The starting time of the movie is ~0.5hr after the placing the SW filter. Scale bar, 500µm.

File Name: Supplementary Movie 3
Description: Body axis morphogenesis of a ruptured SW embryo. Anterior to the left. The starting time of the movie is ~0.5hr after the placing the SW filter. Scale bar, 500µm.

File Name: Supplementary Software 1
Description: LabView codes to control the mechanical probe system and corresponding Matlab codes for data analysis.
