# Supplementary material for: Downregulation of extraembryonic tension controls body axis formation in avian embryos
Source: Nat Commun. 2023 Jun 5;14:3266. doi: 10.1038/s41467-023-38988-3 (PMC10241863; doi:10.1038/s41467-023-38988-3)
Supplement: Supplementary file 7 — Supplementary Software 1 [file 41467_2023_38988_MOESM7_ESM.zip › Kunz_et_al_index_custom_codes.pdf]

Index of Custom Codes

Downregulation of Extraembryonic Tension Controls Body Axis Formation in Avian Embryos

Daniele Kunz[1,2], Anfu Wang[1], Chon U Chan[3], Robyn H. Pritchard[4,5], Wenyu Wang[5], Filomena Gallo[6], Charles R. Bradshaw[1], Elisa Terenzani[1], Karin H. Müller[6], Yan Yan Shery Huang[5], Fengzhu Xiong[1,2,*]

[1]Wellcome Trust / CRUK Gurdon Institute, University of Cambridge, Cambridge, UK
[2]Department of Physiology, Development and Neuroscience, University of Cambridge, Cambridge, UK
[3]Institute of Molecular and Cell Biology, A*STAR, Singapore
[4]Department of Physics, University of Cambridge, Cambridge, UK
[5]Department of Engineering, University of Cambridge, Cambridge, UK
[6]Cambridge Advanced Imaging Centre, University of Cambridge, Cambridge, UK
*correspondence: fx220@cam.ac.uk

Kunz_et_al_codes_mechanical_probe_data_analysis (folder)

Codes for mechanical probe data analysis

Kunz_et_al_mechanical_probe (folder)

LabView codes for the mechanical probe driver
