# Supplementary material for: Downregulation of extraembryonic tension controls body axis formation in avian embryos
Source: Nat Commun. 2023 Jun 5;14:3266. doi: 10.1038/s41467-023-38988-3 (PMC10241863; doi:10.1038/s41467-023-38988-3)
Supplement: Supplementary file 7 — Supplementary Software 1 [file 41467_2023_38988_MOESM7_ESM.zip › Kunz_et_al_mechanical_probe/PT-104 Thermometer/PT-104 dirvers and software development/usbpt104.en.pdf]

# PT-104 Data Logger

User's Guide

usbpt104.en r2
Copyright © 2013 Pico Technology Ltd. All rights reserved.

pico®
Technology

# Contents

1 Introduction ........................................ 1
- 1 Overview ........................................ 1
- 2 Safety warnings ........................................ 2
- 3 Legal information ........................................ 3

2 Product information ........................................ 4
- 1 Pack contents ........................................ 4
- 2 Specifications ........................................ 5
- 3 Installing the driver ........................................ 6
- 4 Connection: USB port ........................................ 6
- 5 Connection: Ethernet port ........................................ 7
- 6 Connection: PRT inputs ........................................ 9
- 7 Setting up ........................................ 10
- 8 The back panel ........................................ 12

3 Background information ........................................ 13
- 1 Platinum resistance thermometers (PRTs) ........................................ 13

Index ........................................ 15

*Copyright © 2013 Pico Technology Ltd. All rights reserved.* 

pico
Technology

# 1 Introduction

## 1.1 Overview

The PT-104 Data Logger is a four-channel, high-resolution temperature converter for use with PT100 and PT1000 type platinum resistance thermometers (PRTs). It can be used to measure temperature, resistance and voltage.

In PT100, PT1000 and resistance modes, the unit can use a two, three or four-wire circuit. Four-wire measurement gives the greatest accuracy.

In voltage mode, the input connector can be treated as a differential input with ground, or as two single-ended inputs.

### Programmer's Guide

All the software that you need for everyday use of the PT-104 Data Logger is supplied with the unit. For more advanced applications, you can write your own software. Information on programming is published in a separate guide:

- PT-104 Data Logger Programmer's Guide (usbpt104pg.en.pdf)

Copyright © 2013 Pico Technology Ltd. All rights reserved.

## 1.2 Safety warnings

We strongly recommend that you read the general safety information below before using your product for the first time. If the equipment is not used in the manner specified then the protection provided may be impaired. This could result in damage to your computer and injury to yourself and others.

DO NOT exceed the maximum input range. The PT-104 Data Logger is designed to measure voltages in the range 0 to +2.5 V. Any voltages in excess of ±30 V may cause permanent damage to the unit.

DO NOT use in contact with mains (line) voltages. This product is not designed for use on circuits carrying mains voltages. Take great care when measuring temperatures near mains (line-powered) equipment. If in doubt, use a meter to check that there is no hazardous AC or DC voltage on the equipment. If a sensor is accidentally connected to a mains voltage, you risk damage to the converter and your computer, and your computer chassis may become live resulting in injury to yourself and others.

DO NOT exceed the maximum input voltage range of the device. Incorrect configuration or use of the device to measure voltages outside this range can be hazardous.

DO NOT rely upon the product's ground connection as a protective safety earth. If there is any possibility of contact with hazardous voltages, the equipment under test must be safely earthed.

DO NOT attempt to repair the unit. The unit contains no user-serviceable parts. Repair or calibration of the unit requires specialised test equipment and must be performed by Pico Technology or its authorised distributors.


Copyright © 2013 Pico Technology Ltd. All rights reserved.

## 1.3 Legal information

The material contained in this release is licensed, not sold. Pico Technology grants a licence to the person who installs this software, subject to the conditions listed below.

Access. The licensee agrees to allow access to this software only to persons who have been informed of these conditions and agree to abide by them.

Usage. The software in this release is for use only with Pico products or with data collected using Pico products.

Copyright. Pico Technology Limited claims the copyright of, and retains the rights to, all material (software, documents etc.) contained in this release. You may copy and distribute the entire release in its original state, but must not copy individual items within the release other than for backup purposes.

Liability. Pico Technology and its agents shall not be liable for any loss, damage or injury, howsoever caused, related to the use of Pico Technology equipment or software, unless excluded by statute.

Fitness for purpose. No two applications are the same: Pico Technology cannot guarantee that its equipment or software is suitable for a given application. It is your responsibility, therefore, to ensure that the product is suitable for your application.

Mission-critical applications. This software is intended for use on a computer that may be running other software products. For this reason, one of the conditions of the license is that it excludes usage in mission-critical applications, for example life support systems.

Viruses. This software was continuously monitored for viruses during production. However, you are responsible for virus-checking the software once it is installed.

Support. If you are dissatisfied with the performance of this software, please contact our technical support staff, who will try to fix the problem within a reasonable time. If you are still dissatisfied, please return the product and software to your supplier within 14 days of purchase for a full refund.

Upgrades. We provide upgrades, free of charge, from our web site at www.picotech.com. We reserve the right to charge for updates or replacements sent out on physical media.

Trademarks. Pico Technology Limited, PicoScope and PicoLog, are trademarks of Pico Technology, registered in the United Kingdom and other countries. Windows XP, Windows Vista and Windows 7 are trademarks of Microsoft Corporation.

*Copyright © 2013 Pico Technology Ltd. All rights reserved.* 

# 2 Product information

## 2.1 Pack contents

Please check that your PT-104 pack (order code PP682) contains the following items:

| Quantity | Reorder code | Description |
|---|---|---|
| 1 | PP682 | PT-104 Data Logger |
| 1 | DO112 | USB Installation Guide |
| 1 | MI106 | USB lead |
| 1 | DI025 | Software and Reference CD |
| 1 | TA076 | Ethernet patch cable |


Copyright © 2013 Pico Technology Ltd. All rights reserved.

## 2.2 Specifications

| | Temperature | Resistance | Voltage |
|---|---|---|---|
| Sensor | PT100*, PT1000 | N/A | N/A |
| Range | -200 to +800 °C | 0 to 375 Ω*<br>0 to 10 kΩ | 0 to 115 mV<br>0 to 2.5 V* |
| Linearity | 20 ppm | 20 ppm | 20 ppm |
| Accuracy @25°C | 0.01 °C* | 20 ppm* | 0.2% (0 to 2.5 V)<br>2% (0 to 115 mV) |
| Temperature coefficient | 5 ppm/°C | 5 ppm/°C | 100 ppm/°C |
| RMS Noise (using filter) | 0.01 °C | 10 ppm | 10 ppm |
| Resolution | 0.001 °C | 1 μΩ | 0.156 μV |
| Conversion Time Per Channel | 720 ms | | |
| Number of inputs | 4 | | |
| Connectors | 4-pin mini-DIN | | |
| Input impedance | >> 1 MΩ | | |
| Overvoltage protection | ±30 V | | |
| Output | USB or Ethernet | | |
| Power | Powered by USB or Ethernet:<br>USB 1.1: 5 V ±10% @ <100 mA<br>USB 2.0: 5 V ±10% @ <200 mA<br>Ethernet: 48 V ±20% @ <40 mA (< 2 W) | | |
| Temperature range | 20 °C to 30 °C for stated accuracy<br>0 °C to 70 °C operating<br>-20 to +80 °C storage | | |
| Humidity range | 20% to 90% RH non-condensing, operating<br>5% to 95% RH non-condensing, storage | | |
| Dimensions | W 135 x L 184 x H 36 mm<br>(5.31 x 7.24 x 1.42 in) | | |
| Software | PicoLog data logging software.<br>Drivers for Windows XP SP3/Windows Vista/Windows 7/ Windows 8.<br>Examples for C/C++, Excel, LabVIEW. | | |
| Ethernet port | Conforms to IEEE 802.3 10Base-T.<br>Compatible with 10/100/1000Base-T networks.<br>Conforms to IEEE 802.3af Power-over-Ethernet (PoE). | | |
| USB port | Conforms to USB 2.0 Full-Speed (12 Mbps) | | |
| *Quoted accuracy is for options marked | | | |

Copyright © 2013 Pico Technology Ltd. All rights reserved.

## 2.3 Installing the driver

The driver is installed automatically when you install the PicoLog software. Alternatively, you can download the driver from our website at http://www.picotech.com.

## 2.4 Connection: USB port

To use the PT-104 Data Logger, connect its USB port to a USB port on your computer using the cable provided.

The first time you connect the device, Windows will install the driver and then tell you that the device is ready for use. You can then run the PicoLog software to start making measurements.


Copyright © 2013 Pico Technology Ltd. All rights reserved.

## 2.5 Connection: Ethernet port

### Setup utility

Before connecting to the PT-104 Data Logger's Ethernet port, you must first configure the Ethernet settings. Proceed as follows:

1. Connect the unit to your computer using the USB port.
2. Run the *Ethernet Settings* utility. There is a shortcut to this utility in the *Pico Technology* program group under the Windows *Start* menu.
3. Set the *Device Types* control to "PT-104 (USB)".
4. The serial number of your device should appear in the device list. Click the device once to select it.
5. Set the *Enable Ethernet Settings* check box.
6. Enter the IP address and port that you wish to give the device. You may need to ask your network manager for an IP address that does not conflict with existing devices on the network.

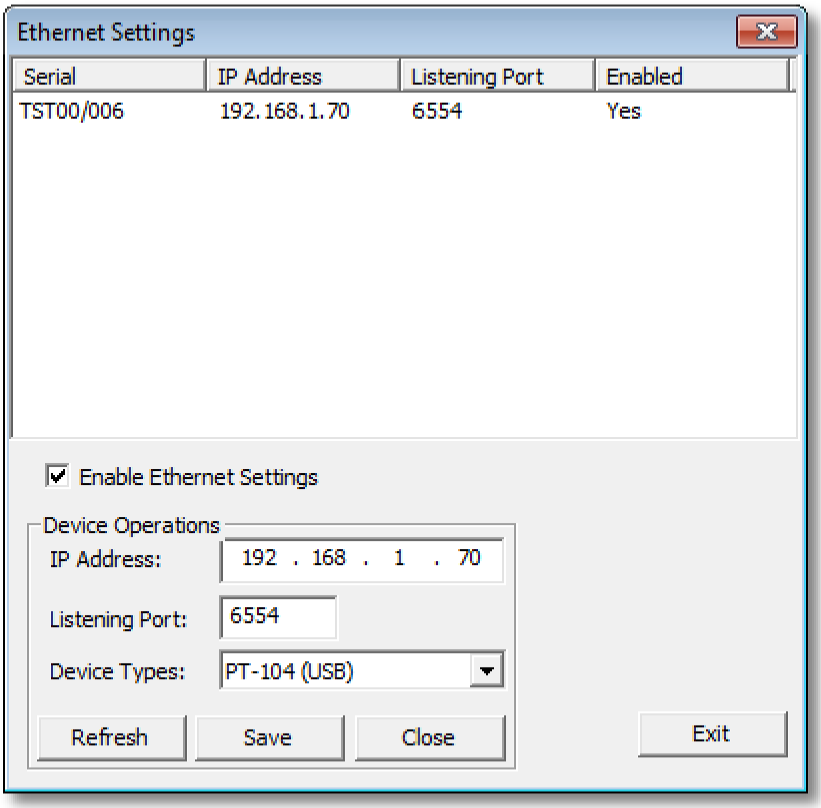


7. Click *Save.*
8. You can now either continue to use the PT-104 Data Logger as a USB device, or unplug the USB cable and reconnect the device using its Ethernet port.

*Copyright © 2013 Pico Technology Ltd. All rights reserved.* 

### Power-saving tip

If you wish to stop using the PT-104 Data Logger in Ethernet mode, you can use the *Ethernet Settings* utility to disable its Ethernet port. This reduces the power consumption of the device when used in USB mode.

### Ethernet and USB

Do not use the PT-104 Data Logger in USB mode when it is being powered by Power-over-Ethernet (PoE). This could cause the unit to radiate electromagnetic energy outside the limits permitted by FCC and EU regulations, and could also cause it to produce spurious measurements. It will not, however, cause permanent damage to the unit.

### LAN connection

To use the PT-104 Data Logger on a local-area network (LAN), connect it to your network switch or network router using the Ethernet cable provided.

### Direct connection

You can also connect the PT-104 Data Logger directly to the network port on your computer. For this connection, you will need to use an Ethernet crossover cable (not supplied).

### Power over Ethernet (PoE)

The PT-104 Data Logger can obtain its power from the Ethernet port as a Powered Device (PD) according to the PoE standard. To use this feature, you must connect the unit to Power Sourcing Equipment (PSE) such as a network switch, router or power injector that also supports the PoE standard. Any standard Ethernet cable up to 100 m (about 328 ft) in length can be used.


*Copyright © 2013 Pico Technology Ltd. All rights reserved.*

## 2.6 Connection: PRT inputs

The PT-104 Data Logger is compatible with PT100 and PT1000 type platinum resistance thermometers (PRTs). Connect each one of these devices to the unit as follows.

Pin Connections to the PT-104 Data Logger Mini-DIN socket

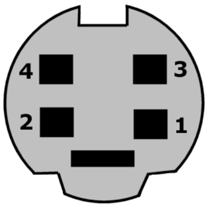


| Pin | PT100, PT1000 or Resistance 4-Wire | PT100, PT1000 or Resistance 3-Wire | PT100, PT1000 or Resistance 2-Wire | Differential voltage |
|---|---|---|---|---|
| 1 | White | Connect to pin 3 | Connect to pin 3 | Do not connect |
| 2 | Red | Red | Red | V - |
| 3 | White | White | White | V + |
| 4 | Red | Red | Connect to pin 2 | Gnd |

Single-ended voltage connection

Single-ended mode allows you to double the number of channels from 4 to 8. It is supported by the driver, so you can use it in your own applications. It is not, however, supported by the PicoLog software.

| Connector | Channel | Pin |
|---|---|---|
| 1 | 1 | 3 |
| | 5 | 2 |
| 2 | 2 | 3 |
| | 6 | 2 |
| 3 | 3 | 3 |
| | 7 | 2 |
| 4 | 4 | 3 |
| | 8 | 2 |

Note on Differential Voltage Mode

The maximum input voltage range of the PT-104 Data Logger is 2.5 V. Any voltage in excess of ±30 V on any input pin may cause permanent damage to the unit.

In Differential Voltage Mode, the input connector should be treated as a differential input with reference to ground. Both inputs (V+ and V-) must be zero volts or above (it does not matter which input has the higher voltage) and must remain within the input range. A ground reference connection is also required for correct operation. The ground connection of each mini-DIN socket consists of a 100 Ω resistor to mains earth/ground through the USB cable outer braiding and the PC chassis.

Copyright © 2013 Pico Technology Ltd. All rights reserved.

## 2.7 Setting up

Set up the PT-104 Data Logger as follows:

1. From the File menu in the PicoLog software Recorder, select New settings. The Recording dialog box appears:

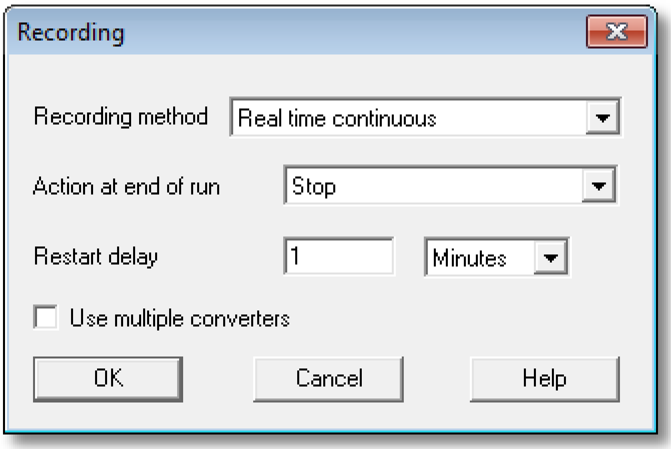


2. Click OK. The Sampling Rate dialog box appears:

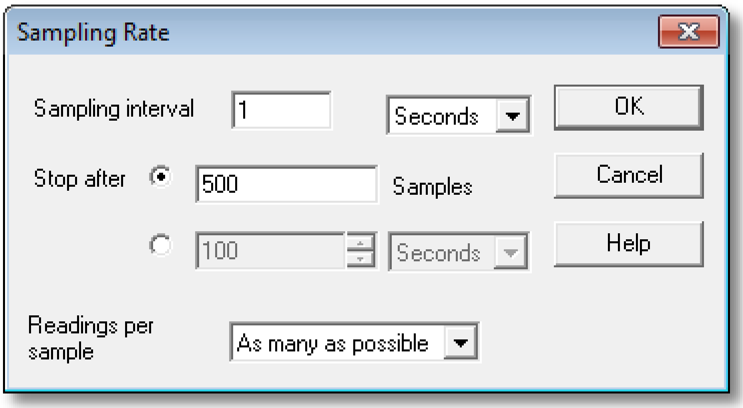


3. Click OK. The Converter details dialog box appears.

4. From the Converter type drop-down list, select either PT-104 (USB) or PT-104 (Ethernet). The dialog box will then show the converter list, as in the following examples:

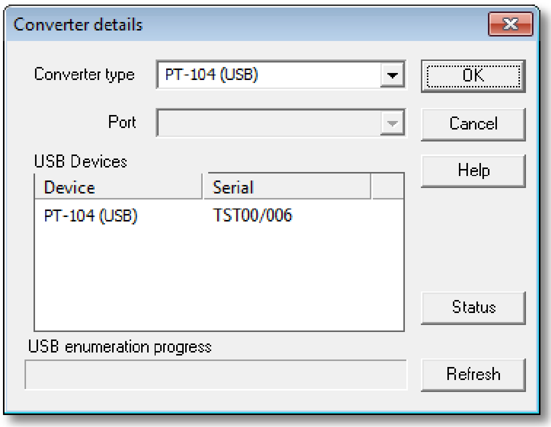


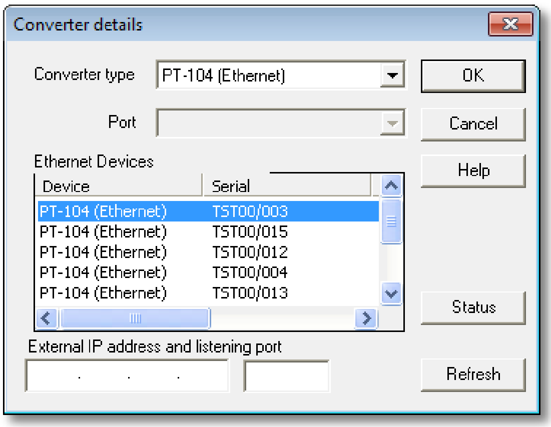



*Copyright © 2013 Pico Technology Ltd. All rights reserved.*

5. USB connection: Select the device with the correct type and serial number, and click OK.

   Ethernet connection: Any PT-104 Data Logger devices visible to your computer on your local network will appear in the list. Select the device with the correct type and IP address, and click OK. If your device does not appear in the list, type its IP address and port number in the boxes below the list.

   When you click OK, the PT-104 Data Logger channels window will appear:

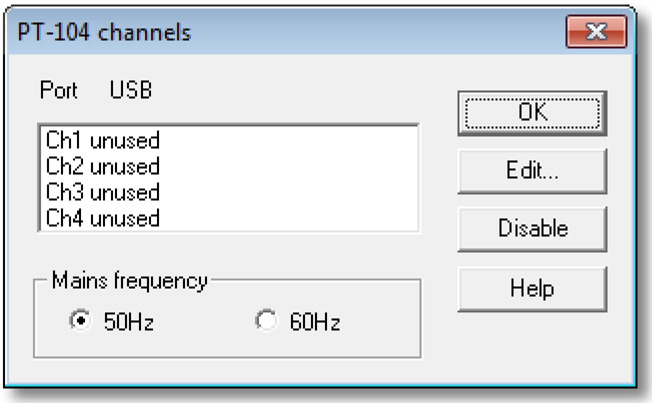


6. In the PT-104 Data Logger channels window, double-click on Ch1 unused. The Edit PT-104 Data Logger Channel dialog box appears:

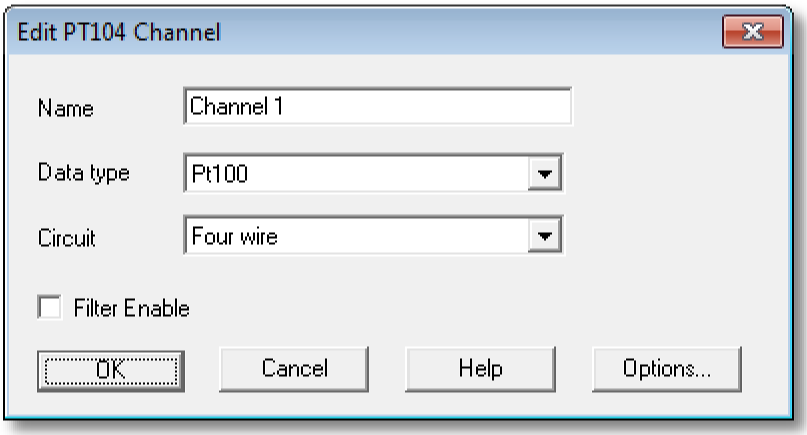


7. Type in a name for the channel, if required.

8. Select the data type required i.e temperature, resistance or voltage.

9. Select circuit - for PT100, PT1000 and Resistance data types only.

*Copyright © 2013 Pico Technology Ltd. All rights reserved.* 

10. Now click OK. Readings from the PT-104 Data Logger should appear in the monitor window:

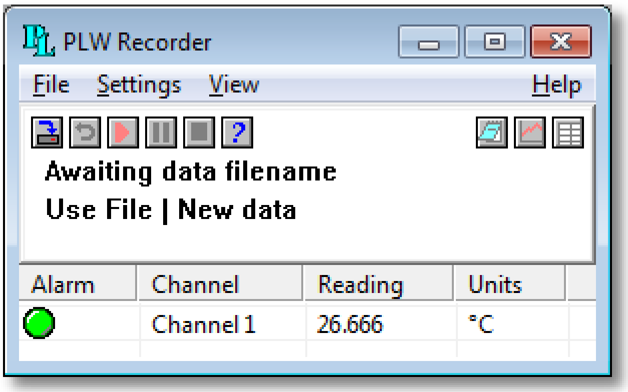


## 2.8 The back panel

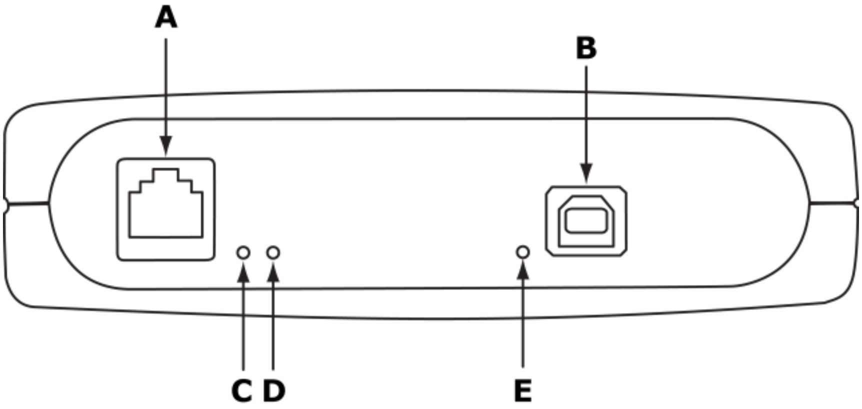


A: Ethernet port

B: USB port

C: Ethernet Data indicator - Flashes to indicate Ethernet data transfer.

D: Ethernet Link indicator - On when Ethernet port connected to an active device.

E: Power/Status indicator - On when power is applied. Flashes to indicate conversion in progress.


Copyright © 2013 Pico Technology Ltd. All rights reserved.

# 3 Background information

## 3.1 Platinum resistance thermometers (PRTs)

PRTs (Platinum Resistance Thermometers) offer excellent accuracy over a wide temperature range (from -200 °C to 850 °C). Sensors are interchangeable between different manufacturers, and are available in various accuracy ratings in packages to suit most applications. Unlike thermocouples, it is not necessary to use special cables to connect to the sensor.

The principle of operation is to measure the resistance of a platinum element. The most common type (PT100) has a resistance of 100 Ω at 0 °C and 138.4 Ω at 100 °C.

The relationship between temperature and resistance is approximately linear over a small temperature range. For example, if you assume that it is linear over the 0 °C to 100 °C range, the error at 50 °C is 0.4 °C. For precision measurement, it is necessary to linearise the resistance to give an accurate temperature. The most recent definition of the relationship between resistance and temperature is International Temperature Standard 90 (ITS-90). This linearisation is done automatically with software.

The linearisation equation is:

$$R_t = R_0 (1 + A \cdot t + B \cdot t^2 + C \cdot (t-100) \cdot t^3)$$

$$A = 3.9083 \times 10^{-3}$$

$$B = -5.775 \times 10^{-7}$$

$$C = \text{(below 0°C) } -4.183 \times 10^{-12}$$

$$\text{(above 0°C) } 0$$

For a PT100 sensor, a 1 °C temperature change will cause a 0.384 Ω change in resistance, so even a small error in measurement of the resistance (for example, the resistance of the wires leading to the sensor) can cause a large error in the measurement of the temperature. For precision work, sensors have four wires - two to carry the sense current, and two to measure the voltage across the sensor element. It is also possible to obtain three-wire sensors, although these operate on the (not necessarily valid) assumption that the resistance of each of the three wires is the same.

The current through the sensor will cause some heating. For example, a sense current of 245 µA through a 100 Ω resistor generates 6 µW of heat. If the sensor element is unable to dissipate this heat, it reports an artificially high temperature. This effect can be reduced by either using a large sensor element, or by making sure that it is in good thermal contact with its environment.

Using a 1 mA sense current gives a signal of only 100 mV. Because the change in resistance for a degree Celsius is very small, even a small error in the measurement of the voltage across the sensor produces a large error in the temperature measurement. For example, a 100 µV voltage measurement error would give a 0.4 °C error in the temperature reading. Similarly, a 1 µA error in the sense current would give a 0.4 °C temperature error.

Copyright © 2013 Pico Technology Ltd. All rights reserved.

Because of the low signal levels, it is important to keep any cables away from electric cables, motors, switchgear and other devices that may emit electrical noise. Using screened cable, with the screen grounded at one end, may help to reduce interference. When using long cables, it is necessary to check that the measuring equipment is capable of handling the resistance of the cables. Most equipment can cope with up to 100 Ω per core.

The type of probe and cable should be chosen carefully to suit the application. The main issues are the temperature range and exposure to fluids (corrosive or conductive) or metals. Clearly, normal solder junctions on cables should not be used at temperatures above about 170 °C.

Sensor manufacturers offer a wide range of sensors that comply with BS1904 class B (DIN 43760). These sensors offer an accuracy of ±0.3 °C at 0 °C. For increased accuracy, you can use BS1904 class A (±0.15 °C) or tenth-DIN sensors (±0.03 °C). Companies like Isotech can provide standards with 0.001 °C accuracy. Please note that these accuracy specifications relate to the SENSOR ONLY. It is necessary to add on any error in the measuring system as well.

Related standards are IEC751 and JISC1604-1989. IEC751 also defines the colour coding for PRT sensor cables: the one or two wires attached to one end of the sensor are red, and the one or two wires at the other end are white.


Copyright © 2013 Pico Technology Ltd. All rights reserved.

# Index


## A

Access 3
Accuracy 5

## B

Back panel 12

## C

Connection
- Ethernet 7
- PRTs 9
- USB 6

Connectors 5
Conversion time 5
Copyright 3

## D

Dimensions 5

## E

Environmental conditions 5
Ethernet
- connection 7
- indicators 12
- port 12

## F

Fitness for purpose 3

## I

Indicators 12
Inputs
- impedance 5
- number of 5

Installation 6

## L

LAN connection 7
Legal information 3
Liability 3
Linearity 5

## M

Mains voltages 2
Maximum input range 2
Mission-critical applications 3

## N

Noise, RMS 5

## O

Output 5
Overview 1
Overvoltage protection 5

## P

Pack contents 4
Power indicator 12
Power-over-Ethernet (PoE) 7
PRT
- background information 13
- connection 9

## R

Range 5
Repairs 2
Resolution 5

## S

Safety warnings 2
Sensor 5
Setting up 10
Software 5
Specifications 5
Status indicator 12
Support 3

## T

Temperature coefficient 5
Trademarks 3

## U

Upgrades 3
Usage 3
USB
- connection 6
- port 12


Copyright © 2013 Pico Technology Ltd. All rights reserved. 

# V

Viruses 3


Copyright © 2013 Pico Technology Ltd. All rights reserved.

Copyright © 2013 Pico Technology Ltd. All rights reserved.

Pico Technology

James House
Colmworth Business Park
ST. NEOTS
Cambridgeshire
PE19 8YP
United Kingdom
Tel: +44 (0) 1480 396 395
Fax: +44 (0) 1480 396 296
www.picotech.com

usbpt104.en r2 26.06.13
Copyright © 2013 Pico Technology Ltd. All rights reserved.
