# Supplementary material for: Downregulation of extraembryonic tension controls body axis formation in avian embryos
Source: Nat Commun. 2023 Jun 5;14:3266. doi: 10.1038/s41467-023-38988-3 (PMC10241863; doi:10.1038/s41467-023-38988-3)
Supplement: Supplementary file 7 — Supplementary Software 1 [file 41467_2023_38988_MOESM7_ESM.zip › Kunz_et_al_mechanical_probe/PT-104 Thermometer/PT-104 dirvers and software development/usbpt104pg.en.pdf]

PT-104 Data Logger

Programmer's Guide

usbpt104pg.en r3
Copyright © 2010-2013 Pico Technology Ltd. All rights reserved.

pico®
Technology

Contents

1 Introduction 1

1 Overview 1

2 Legal information 1

2 Driver information 2

1 Introduction 2

2 Installing the driver 2

3 UsbPt104CloseUnit 2

4 UsbPt104Enumerate 3

5 UsbPt104GetUnitInfo 3

6 UsbPt104GetValue 4

7 UsbPt104IpDetails 5

8 UsbPt104OpenUnit 5

9 UsbPt104OpenUnitViaIp 6

10 UsbPt104SetChannel 7

11 UsbPt104SetMains 7

12 Constants and enumerated types 8

13 Windows 8

3 Writing your own programs 9

1 C 9

2 Excel 9

3 LabVIEW 9

4 Ethernet protocol 10

1 Enabling the Ethernet module 10

2 Finding Ethernet PT-104s 10

3 Commands 10

4 Unlocked unit responses 10

5 Locked unit responses 11

6 To calculate a resistance 11

5 Technical reference 12

1 Lookup table 12

Index 17

Copyright © 2010-2013 Pico Technology Ltd. All rights reserved.

pico®
Technology

1 Introduction

1.1 Overview

The PT-104 is a four-channel, high-resolution data logger for use with PT100 and PT1000 type platinum resistance thermometer (PRT) sensors. As well as temperature, it can also be used to measure resistance and voltage.

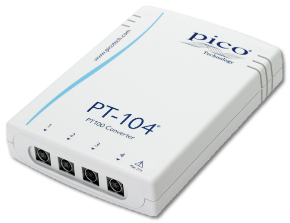

Additional information

For instructions on connecting and using the device, and setting it up with the PicoLog software, please see:

- PT-104 Data Logger User's Guide

1.2 Legal information

The material contained in this release is licensed, not sold. Pico Technology grants a licence to the person who installs this software, subject to the conditions listed below.

Access. The licensee agrees to allow access to this software only to persons who have been informed of these conditions and agree to abide by them.

Usage. The software in this release is for use only with Pico products or with data collected using Pico products.

Copyright. Pico Technology Limited claims the copyright of, and retains the rights to, all material (software, documents etc.) contained in this release. You may copy and distribute the entire release in its original state, but must not copy individual items within the release other than for backup purposes.

Liability. Pico Technology and its agents shall not be liable for any loss, damage or injury, howsoever caused, related to the use of Pico Technology equipment or software, unless excluded by statute.

Fitness for purpose. No two applications are the same: Pico Technology cannot guarantee that its equipment or software is suitable for a given application. It is your responsibility, therefore, to ensure that the product is suitable for your application.

Mission-critical applications. This software is intended for use on a computer that may be running other software products. For this reason, one of the conditions of the license is that it excludes usage in mission-critical applications, for example life support systems.

Viruses. This software was continuously monitored for viruses during production. However, you are responsible for virus-checking the software once it is installed.

Support. If you are dissatisfied with the performance of this software, please contact our technical support staff, who will try to fix the problem within a reasonable time. If you are still dissatisfied, please return the product and software to your supplier within 14 days of purchase for a full refund.

Upgrades. We provide upgrades, free of charge, from our web site at www.picotech.com. We reserve the right to charge for updates or replacements sent out on physical media.

Trademarks. Pico Technology Limited, PicoScope and PicoLog, are trademarks of Pico Technology, registered in the United Kingdom and other countries.

Copyright © 2010-2013 Pico Technology Ltd. All rights reserved.

2 Driver information

2.1 Introduction

The USB PT-104 Software Development Kit contains the drivers and a selection of examples showing how to use them. It also contains a copy of this manual as a PDF file.

The driver routine is supplied as a Windows DLL.

The Windows DLL can be used with C, C++, Delphi and Visual Basic programs: it can also be used with programs like Microsoft Excel, which uses Visual Basic for Applications (VBA) as its macro programming language. More than one application can access the Windows DLL at the same time, as long as the applications do not change the settings for channels that they are not using.

These are the routines in the driver:

- UsbPt104CloseUnit — Close the port (do this each time you finish using the device!)
- UsbPt104Enumerate — Get list of attached devices.
- UsbPt104GetUnitInfo — Get the batch number and serial number, or the calibration date, of this PT-104 Data Logger.
- UsbPt104GetValue — Get the most recent data reading from a channel.
- UsbPt104IpDetails — Read or write IP settings.
- UsbPt104OpenUnit — Open the device through its USB interface.
- UsbPt104OpenUnitViaIp — Open the device through its Ethernet interface.
- UsbPt104SetChannel — Specify the sensor type and filtering for a channel.
- UsbPt104SetMains — Change the mains noise filtering setting to 60 Hz. The default is 50 Hz.

The normal calling sequence for these routines is as follows:

1. Open Driver
2. Set Channels
3. While you want to read data
4. Get data
5. End While
6. Close Unit
7. Close Driver

2.2 Installing the driver

The driver is installed automatically when you install the PicoLog software. Alternatively, you can download the driver from our website at:

http://www.picotech.com/software.

2.3 UsbPt104CloseUnit

```
PICO_STATUS UsbPt104CloseUnit (
    short handle
)
```

This routine disconnects the driver.

Arguments:	handle, identifies the device to close
Returns:	defined in picoStatus.h

Copyright © 2010-2013 Pico Technology Ltd. All rights reserved.

2.4 UsbPt104Enumerate

```
PICO_STATUS UsbPt104Enumerate (
    char                * details,
    unsigned long       * length,
    COMMUNICATION_TYPE type
)
```

This routine returns a list of all the attached PT-104 devices of the specified port type.

Arguments:	details, a string buffer to receive a maximum of length characters<br><br>length, input: the length of the string buffer<br>output: the length of the information string returned<br><br>type, the communication type used by the PT-104. Can be any of the following enumerated types:<br><br>CT_USB = 0x00000001<br>CT_ETHERNET = 0x00000002<br>CT_ALL = 0xFFFFFFFF
Returns:	defined in picoStatus.h

2.5 UsbPt104GetUnitInfo

```
PICO_STATUS  UsbPt104GetUnitInfo (
   short      handle,
   char       * string,
   short      stringLength,
   short      * requiredSize,
   PICO_INFO info
)
```

This routine obtains information on a specified device.

Arguments:	handle, identifies the device whose information is required<br><br>string, output: the information requested<br><br>stringLength, input: the length of the string buffer<br><br>requiredSize, output: the length of the information string requested. If this is longer than stringLength then only the first stringLength characters of the requested information are written to string.<br><br>info, the type of information required. The following types are defined in picoStatus.h:<br><br>PICO_DRIVER_VERSION<br>PICO_USB_VERSION<br>PICO_HARDWARE_VERSION<br>PICO_VARIANT_INFO<br>PICO_BATCH_AND_SERIAL<br>PICO_CAL_DATE<br>PICO_KERNEL_DRIVER_VERSION
Returns:	defined in picoStatus.h

Copyright © 2010-2013 Pico Technology Ltd. All rights reserved.

2.6 UsbPt104GetValue

```
PICO_STATUS UsbPt104GetValue (
  short               handle,
  USBPT104_CHANNELS   channel,
  long                * value,
  short               filtered
)
```

Once you open the driver and define some channels, the driver begins to take continuous readings from the PT-104. When you call this routine, it immediately sets data to the most recent reading for the specified channel.

The scaling of measurements is as follows:

Range	Scaling
Temperature	value × 1/1000 °C
Voltage (0 to 2.5 V)	value × 10 nV
Voltage (0 to 115 mV)	value × 1 nV
Resistance	value × 1 mΩ

Arguments:	handle, identifies the device from which to get data<br><br>channel, the number of the channel to read, from 1 to 4 in differential mode or 1 to 8 in single-ended mode.<br><br>value, output: an array where the sample values will be stored<br><br>filtered, if set to TRUE, the driver returns a low-pass filtered value of the temperature. The time constant of the filter depends on the channel parameters as set by UsbPt104SetChannel, and on how many channels are active.
Returns:	defined in picoStatus.h

Copyright © 2010-2013 Pico Technology Ltd. All rights reserved.

2.7 UsbPt104IpDetails

```
PICO_STATUS UsbPt104IpDetails (
  short                handle,
  short              * enabled,
  char               * ipaddress,
  unsigned short     * length,
  unsigned short     * listeningPort,
  IP_DETAILS_TYPE      type
)
```

This routine either reads or writes the the IP details of a specified device. The `type` argument controls whether the operation is a read or a write.

Arguments:	`handle,` identifies the device that is the target of the operation<br><br>`enabled,` input: 1 to enable the device, 0 to disable<br>output: 1 if the device is enabled, 0 if disabled<br><br>`ipaddress,` input or output: the IP address of the device<br><br>`length,` input or output: the length of the IP address string<br><br>`listeningPort,` input or output: the local IP port connected to the device<br><br>`type,` the type of operation to be performed. Can be either of the following types:<br><br>`IDT_GET,` to read information from the driver<br>`IDT_SET,` to write information to the driver
Returns:	defined in `picoStatus.h`

2.8 UsbPt104OpenUnit

```
PICO_STATUS UsbPt104OpenUnit (
    short * handle,
    char  * serial
)
```

This routine obtains a handle for the PT-104 device with the given serial number.

If you wish to use more than one PT-104, you must call the routine once for each device.

Arguments:	`handle,` output: handle of the device that was opened. This value is used to identify the device in all further function calls.<br><br>`serial,` input: serial number string of device, null-terminated.
Returns:	defined in `picoStatus.h`

Copyright © 2010-2013 Pico Technology Ltd. All rights reserved.

2.9 UsbPt104OpenUnitViaIp

```
PICO_STATUS UsbPt104OpenUnitViaIp (
    short * handle,
    char  * serial,
    char  * ipAddress
)
```

This routine obtains a handle for the Ethernet-connected PT-104 device, identified by either its IP address or its serial number.

- Using IP address identification, a device anywhere on the internet or local network can be opened.
- Using serial number identification, only a device on the local network can be opened.

If you wish to use more than one PT-104, you must call the routine once for each device.

To control the device directly through the Ethernet port without using the usbpt104 DLL, see Ethernet protocol.

Arguments:	`handle,` output: handle of the device that was opened. This value is used to identify the device in all further function calls.<br><br>`serial,` input: serial number of device as a null-terminated string, or a null pointer if `ipAddress` is used<br><br>`ipAddress,` input: the IP address of the device as a null-terminated string, or a null pointer if `serial` is used
Returns:	defined in `picoStatus.h`

Copyright © 2010-2013 Pico Technology Ltd. All rights reserved.

2.10 UsbPt104SetChannel

```
PICO_STATUS UsbPt104SetChannel (
   short                handle,
   USBPT104_CHANNELS    channel,
   USBPT104_DATA_TYPES  type,
   short                noOfWires
)
```

This routine configures a single channel of the specified PT-104. It can be called any time after calling UsbPt104OpenUnit.

The fewer channels selected, the more frequently they will be updated. Measurement takes about 1 second per active channel.

If a call to UsbPt104SetChannel has a type of single-ended, then the specified channel's 'sister' channel is also enabled. For example, enabling 3 also enables 7.

Arguments:	handle, identifies the device to be configured<br><br>channel, which channel you want to set the details for. It should be between 1 and 4 if using single-ended inputs in voltage mode.<br><br>type, the type of reading you require. Choose from the table below.<br><br>noOfWires, how many wires the PT100 or PT1000 sensor has (2, 3 or 4)
Returns:	defined in picoStatus.h

USBPT104_DATA_TYPES		Data type
USBPT104_OFF	0	disable channel
USBPT104_PT100	1	PT100
USBPT104_PT1000	2	PT1000
USBPT104_RESISTANCE_TO_375R	3	resistance 0 to 500 Ω
USBPT104_RESISTANCE_TO_10K	4	resistance 0 to 10 kΩ
USBPT104_DIFFERENTIAL_TO_115MV	5	differential voltage 0 to 100 mV
USBPT104_DIFFERENTIAL_TO_2500MV	6	differential voltage 0 to 2.5 V
USBPT104_SINGLE_ENDED_TO_115MV	7	single-ended voltage 0 to 100 mV
USBPT104_SINGLE_ENDED_TO_2500MV	8	single-ended voltage 0 to 2.5 V

2.11 UsbPt104SetMains

```
PICO_STATUS UsbPt104SetMains (
   short          handle,
   unsigned short sixty_hertz
)
```

This routine is used to inform the driver of the local mains (line) frequency. This helps the driver to filter out electrical noise.

Arguments:	handle, identifies the device to be configured<br><br>sixty_hertz, for 50 Hz set to 0; for 60 Hz set to 1
Returns:	defined in picoStatus.h

Copyright © 2010-2013 Pico Technology Ltd. All rights reserved.

2.12 Constants and enumerated types

```
#define USBPT104_MIN_WIRES 2
#define USBPT104_MAX_WIRES 4

typedef enum enUsbPt104Channels
{
  USBPT104_CHANNEL_1 = 1,
  USBPT104_CHANNEL_2,
  USBPT104_CHANNEL_3,
  USBPT104_CHANNEL_4,
  USBPT104_CHANNEL_5,
  USBPT104_CHANNEL_6,
  USBPT104_CHANNEL_7,
  USBPT104_CHANNEL_8,
   USBPT104_MAX_CHANNELS = USBPT104_CHANNEL_8
} USBPT104_CHANNELS;

typedef enum enUsbPt104DataType
{
   USBPT104_OFF,
   USBPT104_PT100,
   USBPT104_PT1000,
   USBPT104_RESISTANCE_TO_375R,
   USBPT104_RESISTANCE_TO_10K,
   USBPT104_DIFFERENTIAL_TO_115MV,
   USBPT104_DIFFERENTIAL_TO_2500MV,
   USBPT104_SINGLE_ENDED_TO_115MV,
   USBPT104_SINGLE_ENDED_TO_2500MV,
   USBPT104_MAX_DATA_TYPES
} USBPT104_DATA_TYPES;

typedef enum enIpDetailsType
{
   IDT_GET,
   IDT_SET,
} IP_DETAILS_TYPE;

typedef enum enCommunicationType
{
   CT_USB = 0x00000001,
   CT_ETHERNET = 0x00000002,
   CT_ALL = 0xFFFFFFFF
} COMMUNICATION_TYPE;
```

2.13 Windows

The 32-bit Windows driver is the file `usbpt104.dll`, which is included in the SDK. If an application is unable to find the DLL, try moving the DLL to `c:\windows\system`.

Copyright © 2010-2013 Pico Technology Ltd. All rights reserved.

3 Writing your own programs

3.1 C

The C example is a console mode program that demonstrates the facilities of the driver.

To compile the program, create a new project containing the following files from the USB PT-104 SDK:

- `USBPT104con.c`

and:

- `UsbPt104bc.lib` (Borland 32-bit applications) or
- `UsbPt104.lib` (Microsoft Visual C 32-bit applications)

The following file must be in the compilation directory:

- `UsbPt104Api.h`

and the following file must be in the same directory as the executable:

- `USBPT104.dll`

3.2 Excel

The easiest way to transfer data into Excel is to use PicoLog.

If, however, you need to do something that is not possible using PicoLog, you can write an Excel macro that calls `USBPT104.dll` to read in a set of data values. The Excel Macro language is similar to Visual Basic.

The example `USBPT104.xls` reads values from all four channels every second and assigns them to cells in the spreadsheet.

3.3 LabVIEW

The routines described here were created using LabVIEW 8.2 on Windows XP.

To use these routines, copy `USBPT104.dll` to your LabVIEW `user.lib` directory.

`USBPT104.vi` is a fully functional example LabVIEW application. It demonstrates how to connect to the device using both USB and Ethernet. It also demonstrates reading all possible measurement types from the four channels.

Copyright © 2010-2013 Pico Technology Ltd. All rights reserved.

4 Ethernet protocol

Using the protocol described below, the PT-104 can be controlled directly through the Ethernet port without using the USBPT104 DLL.

4.1 Enabling the Ethernet module

By default the Ethernet module is disabled to save power. To enable it, plug the connector into a USB port and use the Ethernet settings application installed with PicoLog. Once an IP address and port are assigned, the module will be enabled. The unit may then be used by powering from USB or PoE.

4.2 Finding Ethernet PT-104s

To discover all PT104 data loggers on a network:

- Send a UDP packet to port 23 from port 23 (telnet) to destination 255.255.255.255 and data fff - 0x666666.
- Replies from all PT104s will be to the IP address requested from and to port 23 from 23. The data will be PT104 Mac:xxxxxx Lock:Y Port:ZZ, where
  xxxxxx is the 6 byte MAC address of the PT104 replying and Y is 0x00 for unlocked and 0x01 for locked, and
  ZZ is the port it will listen on (in hex MSB first).

4.3 Commands

- To lock a PT104 to a machine, a UDP packet destined for the device's listening port and IP address should be sent containing the data lock. This is required before the PT104 is usable.

UDP packet data in the form Command + data bytes

Command	Data bytes	Function
0x30	0x00 for 50 Hz<br>Any other byte for 60 Hz	Change mains frequency rejection
0x31	One byte, bit 0 is LSB<br>Bit 0: enable channel 1<br>Bit 1: enable channel 2<br>Bit 2: enable channel 3<br>Bit 3: enable channel 4<br>Bit 4: channel 1 gain<br>Bit 5: channel 2 gain<br>Bit 6: channel 3 gain<br>Bit 7: channel 4 gain	Start converting<br><br>enable:<br>0 - off<br>1 - on<br><br>gain:<br>0 - x1<br>1 - x21 (for 375 Ω range)
0x32	-	Read Eprom
0x33	-	Unlock
0x34	-	Keep alive

Unlock / timeout stops the transfer of any channel data. Unlock should be used when PicoLog no longer requires the unit so that it is unlocked for use with other machines.

4.4 Unlocked unit responses

- PT104 Mac:XXXXXX Lock:Y Port:ZZ

This is the only response possible until a lock and can be obtained with a discovery packet only.

Copyright © 2010-2013 Pico Technology Ltd. All rights reserved.

4.5 Locked unit responses

- Lock Success
- Lock Success (already locked to this machine)
- Unlocked
- Eeprom={byte[128]}
- Converting
- Mains Changed
- Unknown Command
- Alive
- [hex]00XXXX01XXXX02XXXX03XXXX data from channel 1
- [hex]04XXXX05XXXX06XXXX07XXXX data from channel 2
- [hex]08XXXX09XXXX0aXXXX0bXXXX data from channel 3
- [hex]0cXXXX0dXXXX0eXXXX0fXXXX data from channel 4
  (this data format is: Measurement 0, 1, 2, 3 for respective channels)

A unit that has been locked will reply *Lock Success* or, if currently locked by this machine, *Lock Success (already locked to this machine).*

Converting is an acknowledgment of any convert request received. Once a setting has been made the unit will continue to convert and send back data. This means there will be incoming data approximately every 720ms. To stop all converting, send a converting command with data *0x00* to turn off all channels.

After receiving the lock command via UDP, a 15 s timeout will start and a keep-alive packet will need to be sent in this time. The *Alive* response is the acknowledgment. The unit should try to be kept alive each 10 seconds to allow time to retry if no acknowledgment is received.

If any request is made from another machine while a unit is locked then the response will be *PT104 Mac:XXXXXX Lock:Y Port:ZZ* as above.

EEPROM FORMAT

Index	NumBytes	Data
0	19	Reserved
19	10	Batch
29	8	Calibration Date
37	4	Ch1 Calibration
41	4	Ch2 Calibration
45	4	Ch3 Calibration
49	4	Ch4 Calibration
53	6	MAC Address
59	67	Reserved
126	2	Checksum

4.6 To calculate a resistance

- Read the EEPROM to obtain the calibration information for the channels.
- Take measurement 0, 1, 2 and 3 on a channel.
- Then: result = (channel calibration * (measurement 3 - measurement 2)) / (measurement 1 - measurement 0)
  to convert to a resistance divide result by 1,000,000.0.

For other measurement types see the Protocol information in the serial port *PT-104 User's Guide*.

Copyright © 2010-2013 Pico Technology Ltd. All rights reserved.

5 Technical reference

5.1 Lookup table

Here is the resistance-temperature characteristic for a PT100 sensor.

Temp (°C)	Resistance (Ω)
-50	80.306282
-49	80.703340
-48	81.100257
-47	81.497036
-46	81.893677
-45	82.290179
-44	82.686545
-43	83.082774
-42	83.478868
-41	83.874827
-40	84.270652
-39	84.666343
-38	85.061901
-37	85.457327
-36	85.852622
-35	86.247785
-34	86.642818
-33	87.037721
-32	87.432495
-31	87.827140
-30	88.221657
-29	88.616046
-28	89.010309
-27	89.404445
-26	89.798455
-25	90.192339
-24	90.586099
-23	90.979734
-22	91.373246
-21	91.766634
-20	92.159898
-19	92.553041
-18	92.946061
-17	93.338960
-16	93.731737
-15	94.124394
-14	94.516930
-13	94.909346
-12	95.301643
-11	95.693820
-10	96.085879
-9	96.477819
-8	96.869641
-7	97.261345
-6	97.652931
-5	98.044401
-4	98.435753
-3	98.826989
-2	99.218109
-1	99.609112
0	100.000000

Copyright © 2010-2013 Pico Technology Ltd. All rights reserved.

1	100.390772
2	100.781429
3	101.171970
4	101.562396
5	101.952706
6	102.342901
7	102.732980
8	103.122944
9	103.512792
10	103.902525
11	104.292142
12	104.681644
13	105.071030
14	105.460301
15	105.849456
16	106.238496
17	106.627420
18	107.016229
19	107.404922
20	107.793500
21	108.181962
22	108.570309
23	108.958540
24	109.346656
25	109.734656
26	110.122541
27	110.510310
28	110.897964
29	111.285502
30	111.672925
31	112.060232
32	112.447424
33	112.834500
34	113.221461
35	113.608306
36	113.995036
37	114.381650
38	114.768149
39	115.154532
40	115.540800
41	115.926952
42	116.312989
43	116.698910
44	117.084716
45	117.470406
46	117.855981
47	118.241440
48	118.626784
49	119.012012
50	119.397125
51	119.782122
52	120.167004
53	120.551770
54	120.936421
55	121.320956
56	121.705376
57	122.089680
58	122.473869

Copyright © 2010-2013 Pico Technology Ltd. All rights reserved.

59	122.857942
60	123.241900
61	123.625742
62	124.009469
63	124.393080
64	124.776576
65	125.159956
66	125.543221
67	125.926370
68	126.309404
69	126.692322
70	127.075125
71	127.457812
72	127.840384
73	128.222840
74	128.605181
75	128.987406
76	129.369516
77	129.751510
78	130.133389
79	130.515152
80	130.896800
81	131.278332
82	131.659749
83	132.041050
84	132.422236
85	132.803306
86	133.184261
87	133.565100
88	133.945824
89	134.326432
90	134.706925
91	135.087302
92	135.467564
93	135.847710
94	136.227741
95	136.607656
96	136.987456
97	137.367140
98	137.746709
99	138.126162
100	138.505500
101	138.884722
102	139.263829
103	139.642820
104	140.021696
105	140.400456
106	140.779101
107	141.157630
108	141.536044
109	141.914342
110	142.292525
111	142.670592
112	143.048544
113	143.426380
114	143.804101
115	144.181706
116	144.559196

Copyright © 2010-2013 Pico Technology Ltd. All rights reserved.

117	144.936570
118	145.313829
119	145.690972
120	146.068000
121	146.444912
122	146.821709
123	147.198390
124	147.574956
125	147.951406
126	148.327741
127	148.703960
128	149.080064
129	149.456052
130	149.831925
131	150.207682
132	150.583324
133	150.958850
134	151.334261
135	151.709556
136	152.084736
137	152.459800
138	152.834749
139	153.209582
140	153.584300
141	153.958902
142	154.333389
143	154.707760
144	155.082016
145	155.456156
146	155.830181
147	156.204090
148	156.577884
149	156.951562
150	157.325125
151	157.698572
152	158.071904
153	158.445120
154	158.818221
155	159.191206
156	159.564076
157	159.936830
158	160.309469
159	160.681992
160	161.054400
161	161.426692
162	161.798869
163	162.170930
164	162.542876
165	162.914706
166	163.286421
167	163.658020
168	164.029504
169	164.400872
170	164.772125
171	165.143262
172	165.514284
173	165.885190
174	166.255981

Copyright © 2010-2013 Pico Technology Ltd. All rights reserved.

175	166.626656
176	166.997216
177	167.367660
178	167.737989
179	168.108202
180	168.478300
181	168.848282
182	169.218149
183	169.587900
184	169.957536
185	170.327056
186	170.696461
187	171.065750
188	171.434924
189	171.803982
190	172.172925
191	172.541752
192	172.910464
193	173.279060
194	173.647541
195	174.015906
196	174.384156
197	174.752290
198	175.120309
199	175.488212
200	175.856000

Copyright © 2010-2013 Pico Technology Ltd. All rights reserved.

Index

B

Batch number 3

C

C 9
Calbration date 3
Channel setup 7
Closing a unit 2
COMMUNICATION_TYPE type 8

D

Driver version 3

E

Ethernet port 6
Excel 9

H

Handle, obtaining 5
Hardware version 3

I

Installation 2
IP address 6
IP details 5
IP_DETAILS_TYPE type 8

K

Kernel driver version 3

L

LabVIEW 9
Legal information 1
Lookup table 12

M

Mains frequency 7

O

Opening a unit 5

S

Serial number 3

U

USB version 3
USBPT104_CHANNELS type 8
USBPT104_DATA_TYPES type 8
USBPT104_MAX_WIRES constant 8
USBPT104_MIN_WIRES constant 8
UsbPt104CloseUnit 2
UsbPt104Enumerate 3
UsbPt104GetUnitInfo 3
UsbPt104GetValue 4
UsbPt104IpDetails 5
UsbPt104OpenUnit 5
UsbPt104OpenUnitViaIp 6
UsbPt104SetChannel 7
UsbPt104SetMains 7

V

Variant information 3

W

Windows 8

Copyright © 2010-2013 Pico Technology Ltd. All rights reserved.

pico®
Technology

Pico Technology

James House
Colmworth Business Park
ST. NEOTS
Cambridgeshire
PE19 8YP
United Kingdom
Tel: +44 (0) 1480 396 395
Fax: +44 (0) 1480 396 296
www.picotech.com

usbpt104pg.en r3 10.05.13
Copyright © 2010-2013 Pico Technology Ltd. All rights reserved.
