# Supplementary material for: Downregulation of extraembryonic tension controls body axis formation in avian embryos
Source: Nat Commun. 2023 Jun 5;14:3266. doi: 10.1038/s41467-023-38988-3 (PMC10241863; doi:10.1038/s41467-023-38988-3)
Supplement: Supplementary file 8 — Reporting Summary [file 41467_2023_38988_MOESM8_ESM.pdf]

nature portfolio

Corresponding author(s): Fengzhu Xiong

Last updated by author(s): May 12, 2023

Reporting Summary

Nature Portfolio wishes to improve the reproducibility of the work that we publish. This form provides structure for consistency and transparency in reporting. For further information on Nature Portfolio policies, see our Editorial Policies and the Editorial Policy Checklist.

Statistics

For all statistical analyses, confirm that the following items are present in the figure legend, table legend, main text, or Methods section.

n/a	Confirmed	
☐	☒	The exact sample size (*n*) for each experimental group/condition, given as a discrete number and unit of measurement
☐	☒	A statement on whether measurements were taken from distinct samples or whether the same sample was measured repeatedly
☐	☒	The statistical test(s) used AND whether they are one- or two-sided<br>*Only common tests should be described solely by name; describe more complex techniques in the Methods section.*
☒	☐	A description of all covariates tested
☐	☒	A description of any assumptions or corrections, such as tests of normality and adjustment for multiple comparisons
☐	☒	A full description of the statistical parameters including central tendency (e.g. means) or other basic estimates (e.g. regression coefficient) AND variation (e.g. standard deviation) or associated estimates of uncertainty (e.g. confidence intervals)
☐	☒	For null hypothesis testing, the test statistic (e.g. F, t, r) with confidence intervals, effect sizes, degrees of freedom and P value noted<br>*Give P values as exact values whenever suitable.*
☒	☐	For Bayesian analysis, information on the choice of priors and Markov chain Monte Carlo settings
☒	☐	For hierarchical and complex designs, identification of the appropriate level for tests and full reporting of outcomes
☒	☐	Estimates of effect sizes (e.g. Cohen's d, Pearson's r), indicating how they were calculated

Our web collection on statistics for biologists contains articles on many of the points above.

Software and code

Policy information about availability of computer code

Data collection	Zen LITE, 2020 (Carl Zeiss), MicroscopeVIS2.0 (Kern), FEI MAPS (1.1.9.605), custom LabView (2019) codes (provided as supplemental software)
Data analysis	FIJI (Image J 1.53t), Excel and Powerpoint (2021, Microsoft), custom Matlab (2020a) codes (provided as supplemental software)

For manuscripts utilizing custom algorithms or software that are central to the research but not yet described in published literature, software must be made available to editors and reviewers. We strongly encourage code deposition in a community repository (e.g. GitHub). See the Nature Portfolio guidelines for submitting code & software for further information.

Data

Policy information about availability of data

All manuscripts must include a data availability statement. This statement should provide the following information, where applicable:

- Accession codes, unique identifiers, or web links for publicly available datasets
- A description of any restrictions on data availability
- For clinical datasets or third party data, please ensure that the statement adheres to our policy

The data generated in this study are provided in the Source Data file accompanying the paper and Supplemental Information

Human research participants

Policy information about studies involving human research participants and Sex and Gender in Research.

Reporting on sex and gender	n/a
Population characteristics	n/a
Recruitment	n/a
Ethics oversight	n/a

Note that full information on the approval of the study protocol must also be provided in the manuscript.

Field-specific reporting

Please select the one below that is the best fit for your research. If you are not sure, read the appropriate sections before making your selection.

☒ Life sciences ☐ Behavioural & social sciences ☐ Ecological, evolutionary & environmental sciences

For a reference copy of the document with all sections, see nature.com/documents/nr-reporting-summary-flat.pdf

Life sciences study design

All studies must disclose on these points even when the disclosure is negative.

Sample size	For quantitative comparisons (e.g., axis length), at least 5 samples per group are used per experiment. Most experiments involve n>10 samples per group. This sample size is in line with the practice of the field (e.g., Oginuma et al., Nature, 2020). This sample size improves consistency and minimizes system errors that might be introduced between groups when total experiment time becomes too long (>2 hrs), taking into consideration the time consuming procedure of embryo preparation (Chapman et al., Dev Dyn, 2001) and individual embryo operations (Xiong et al., Dev Cell, 2020). Replications were performed to confirm the results. Before applying t-tests, the measurements were normalized to the mean and pooled for a chi square goodness of fit test. The tests suggest that the variabilities observed do not come from a distribution significantly different (p>0.05) from a normal distribution.
Data exclusions	NO data point exclusions in any analysis. For in ovo experiments (such as Oil experiments), unfertilized eggs were not included. For in vitro experiments (such as SW experiments), embryos that show unspecific developmental abnormalities upon extraction were discarded. This health screen is applied in an unbiased manner to all experimental and control groups.
Replication	All wet experiments reported have been replicated at least once (key experiments 3 or more times) with a different batch of eggs (shipment in a different week) and new preparation of reagents (culture and imaging plates, injection needles and labeling reagents (different aliquots of the same source)). The same dissecting tools, imaging protocols and microscopes were used during replications.
Randomization	In each embryonic experimental test involving groups of embryos, all embryos were incubated, prepared and handled together except for the particular experimental condition(s) being tested. After an initial health screen of all embryos, the investigators do not use any criteria or variables when allocating embryos into control and experimental groups.
Blinding	Blinding is not possible for embryology perturbations (e.g., SW experiments) on the embryos as data collection and analysis require the investigator to look at the images where the type of perturbation is clearly visible. For VM mechanical measurements, a look-up table (relating experimental conditions to sample number) is generated at the operation time and is not referred to during data collection and analysis. Only when analysis is complete is the look-up table linked to sort the results into different experimental groups. Blinding is not applicable in confocal imaging as the test conditions are clearly affiliated with the data throughout data acquisition and analysis.

Reporting for specific materials, systems and methods

We require information from authors about some types of materials, experimental systems and methods used in many studies. Here, indicate whether each material, system or method listed is relevant to your study. If you are not sure if a list item applies to your research, read the appropriate section before selecting a response.

March 2021

Materials & experimental systems

n/a	Involved in the study
☒	☐ Antibodies
☒	☐ Eukaryotic cell lines
☒	☐ Palaeontology and archaeology
☐	☒ Animals and other organisms
☒	☐ Clinical data
☒	☐ Dual use research of concern

Methods

n/a	Involved in the study
☒	☐ ChIP-seq
☒	☐ Flow cytometry
☒	☐ MRI-based neuroimaging

Animals and other research organisms

Policy information about studies involving animals; ARRIVE guidelines recommended for reporting animal research, and Sex and Gender in Research

Laboratory animals	Chicken (gallus gallus), limited to eggs and early embryos (<7 days) only
Wild animals	no wild animals were used in the study
Reporting on sex	Chicken embryos used in this study are not screened or sorted genetically for sex. Sex is not expected to affect the processes studied
Field-collected samples	no field collected samples were used in the study
Ethics oversight	Not required

Note that full information on the approval of the study protocol must also be provided in the manuscript.
