# Supplementary material for: Downregulation of extraembryonic tension controls body axis formation in avian embryos
Source: Nat Commun. 2023 Jun 5;14:3266. doi: 10.1038/s41467-023-38988-3 (PMC10241863; doi:10.1038/s41467-023-38988-3)
Supplement: Supplementary file 9 — Source Data [file 41467_2023_38988_MOESM9_ESM.zip › Kunz_et_al_AW_stretcher_technical_drawings.pdf]

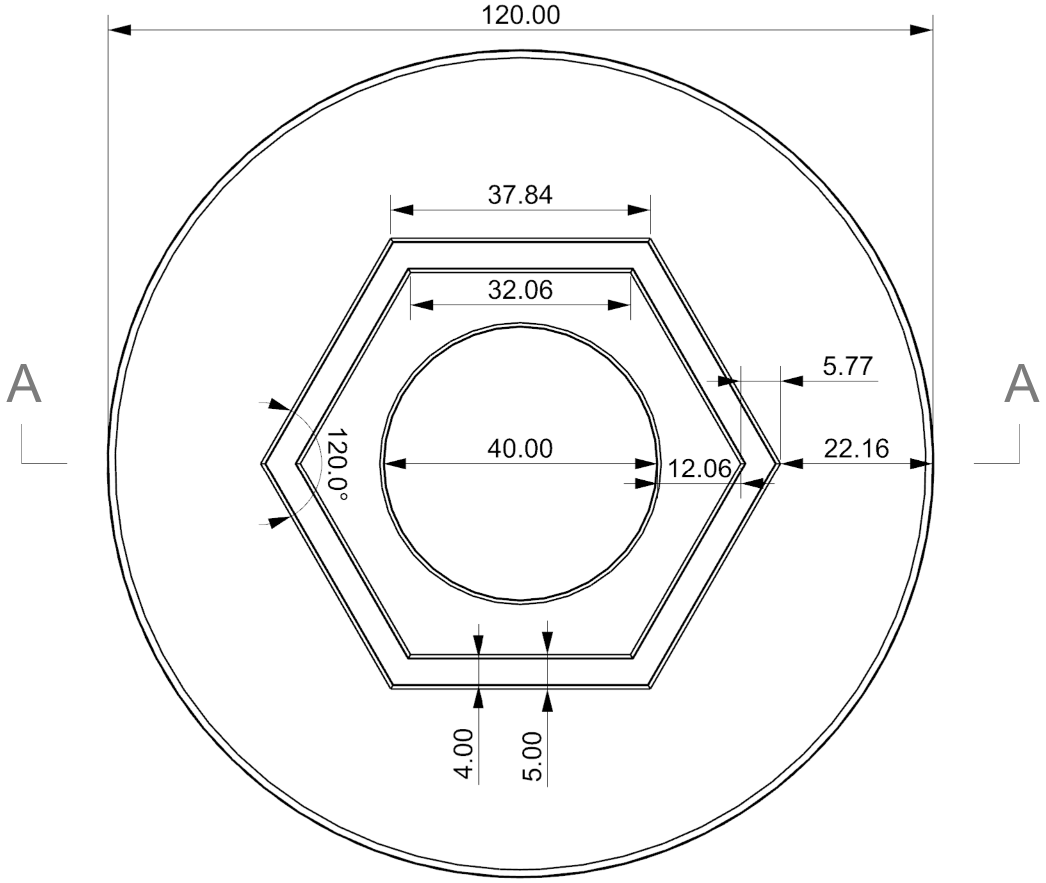

PLAN

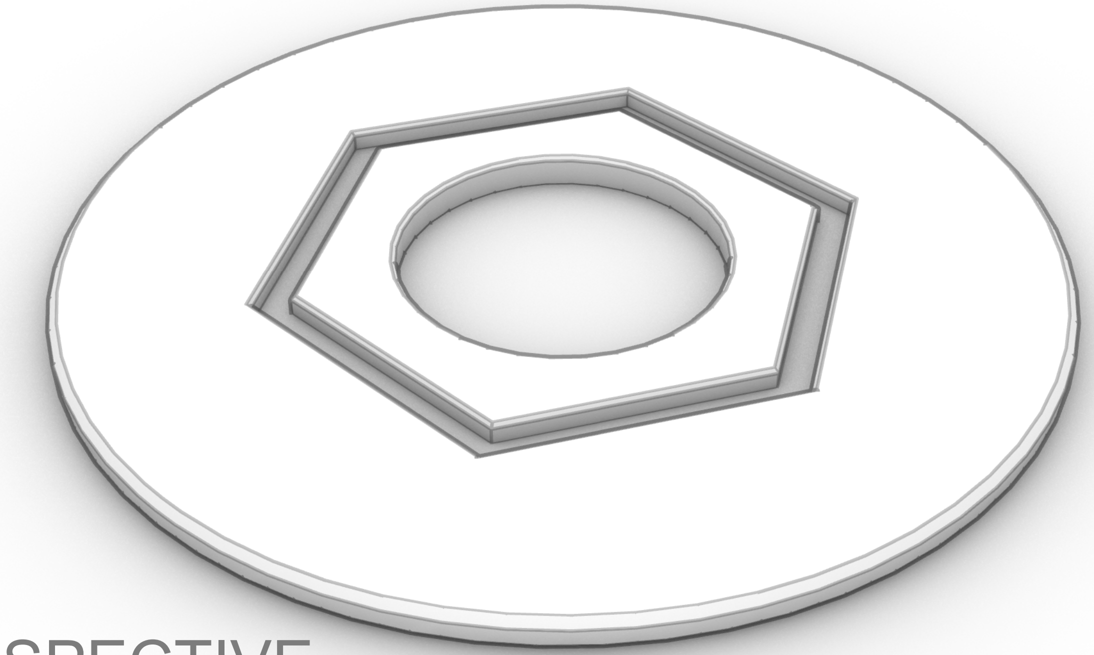

PERSPECTIVE

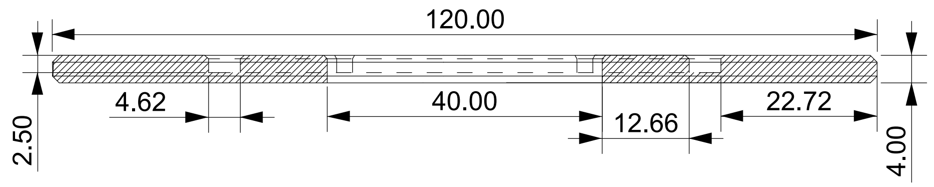

SECTION AA

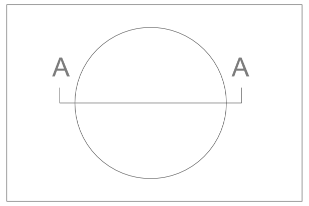

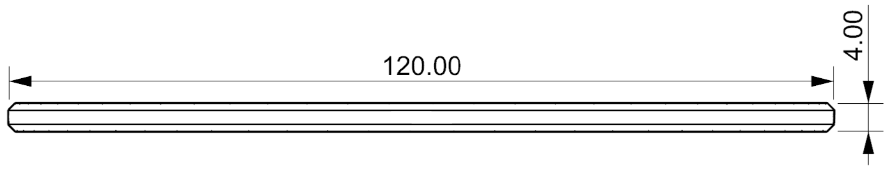

ELEVATION

Title: Technical drawing of the stretcher base

Unit: mm

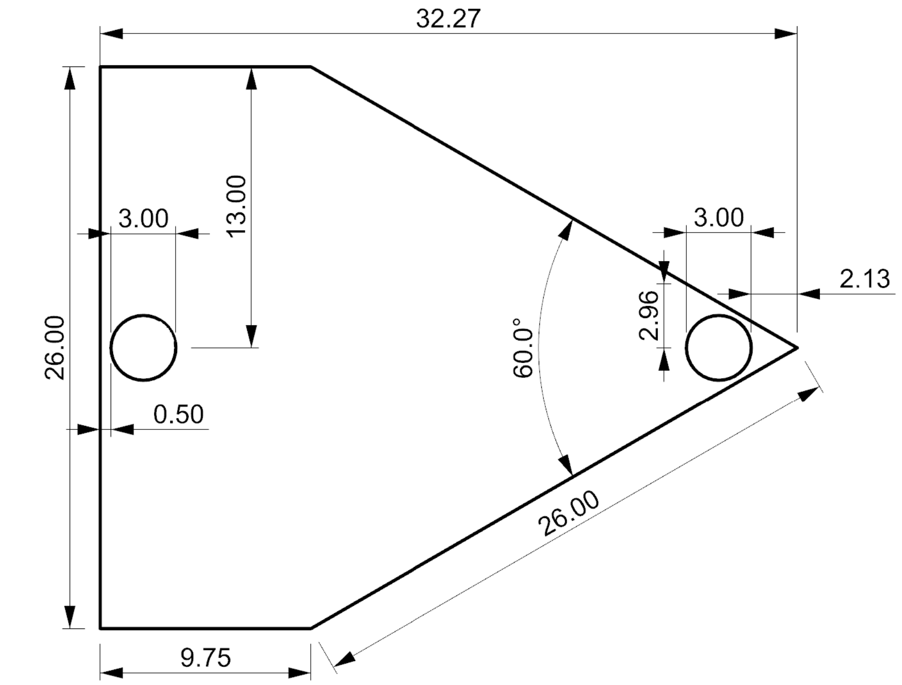

Top

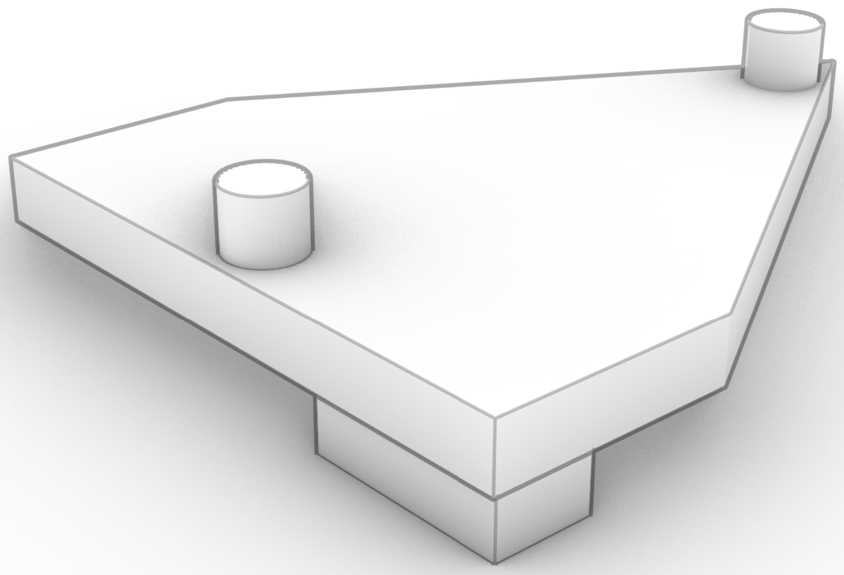

Perspective

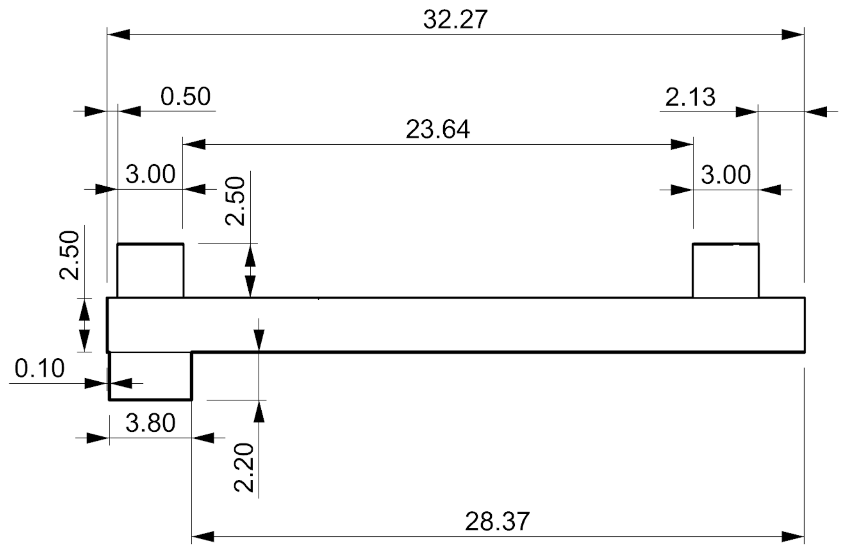

Right

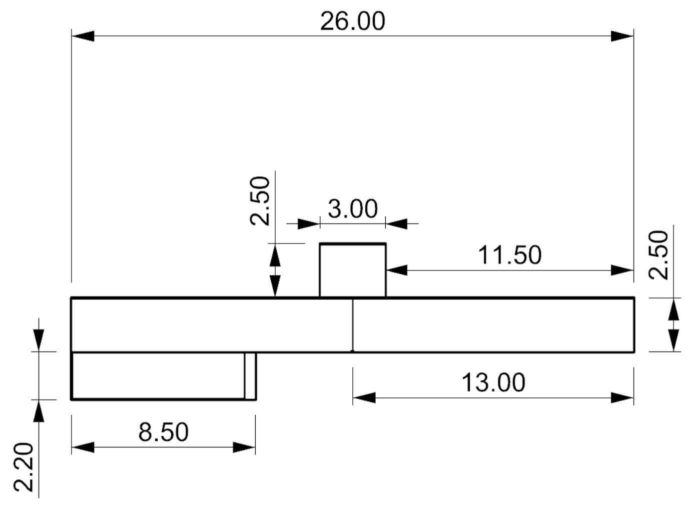

Front

Title: Technical drawing of the stretcher blade

Unit: mm

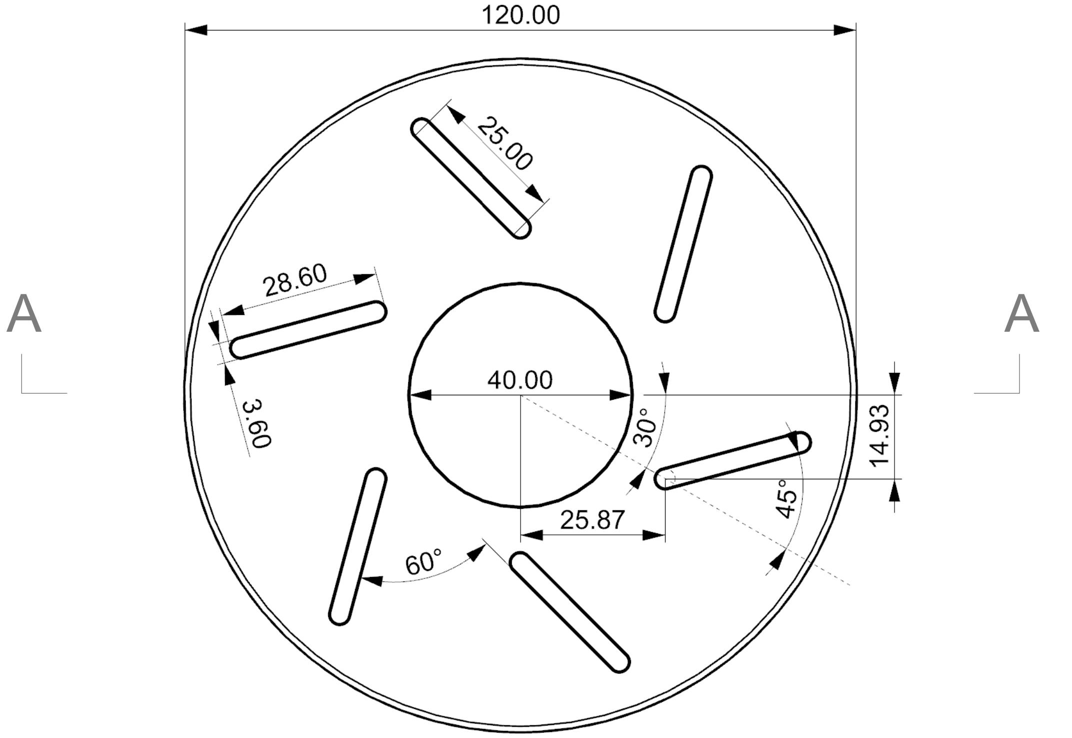

PLAN

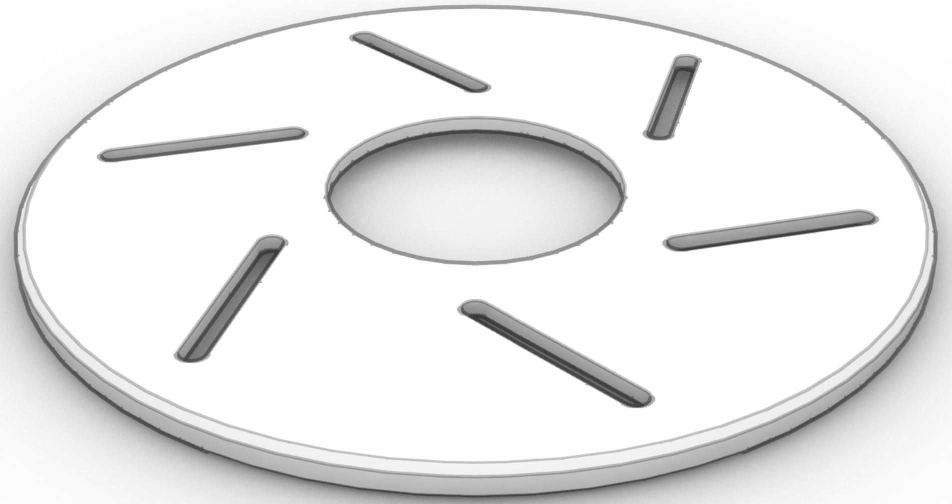

PERSPECTIVE

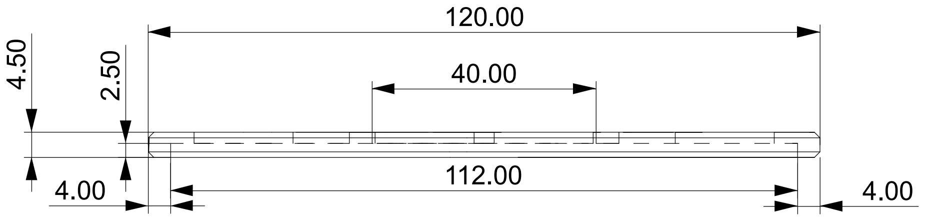

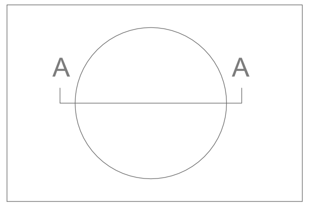

SECTION AA

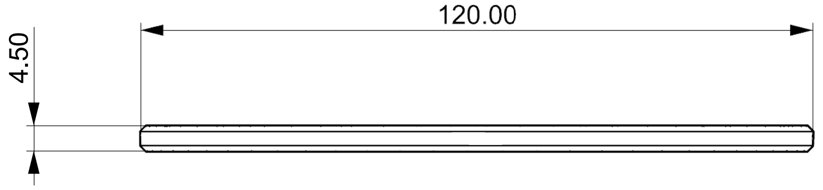

ELEVATION

Title: Technical drawing of the stretcher lid

Unit: mm
