# Supplementary figures and images for: Downregulation of extraembryonic tension controls body axis formation in avian embryos
Source: Nat Commun. 2023 Jun 5;14:3266. doi: 10.1038/s41467-023-38988-3 (PMC10241863; doi:10.1038/s41467-023-38988-3)

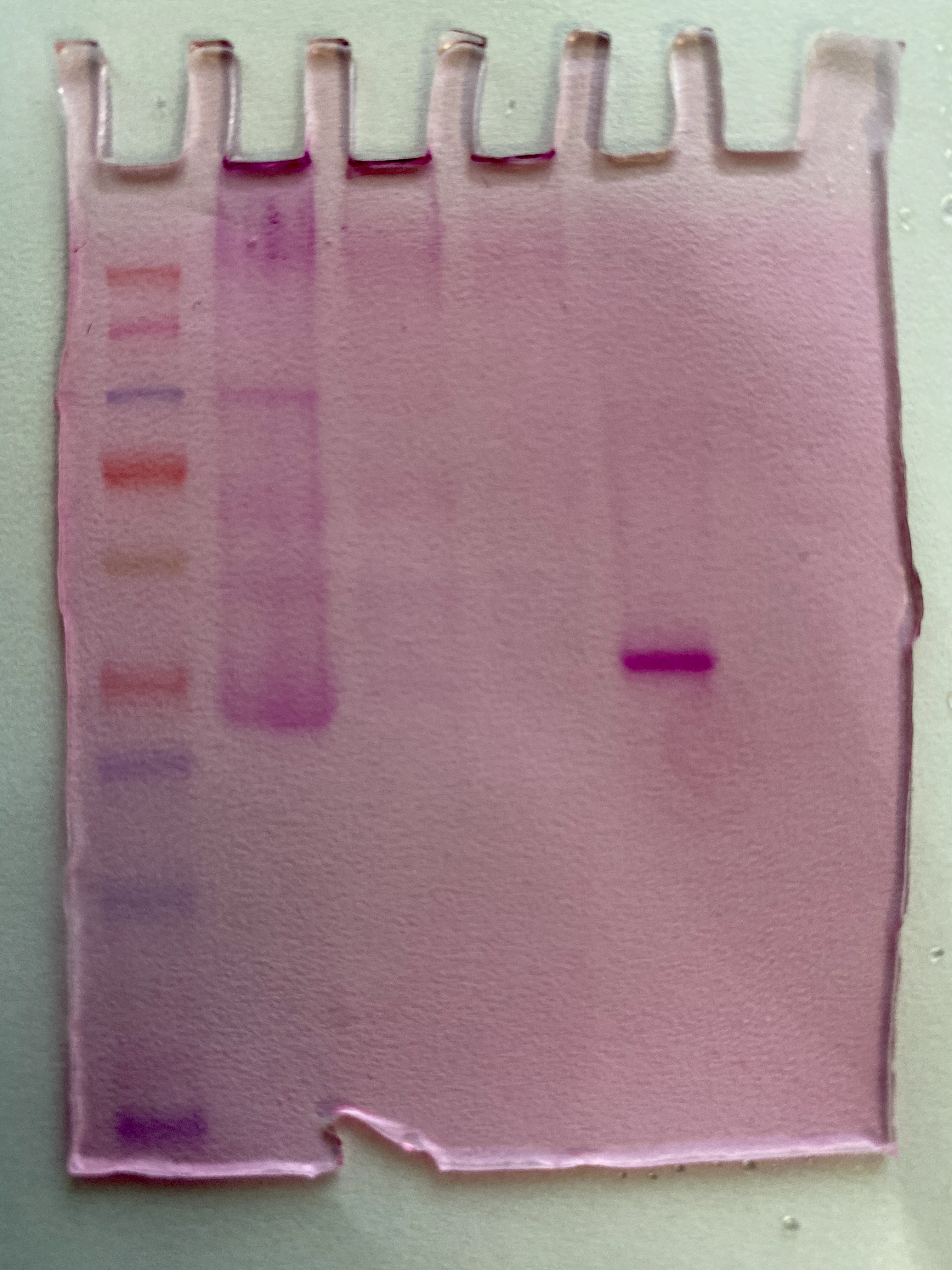

Supplement: Supplementary file 9 — Source Data [file 41467_2023_38988_MOESM9_ESM.zip › Kunz_et_al_gels/IMG_0938.jpg]
